# Decoupling stability and release in disulfide bonds with antibody-small molecule conjugates†
†Electronic supplementary information (ESI) available. See DOI: 10.1039/c6sc01831a



**DOI:** 10.1039/c6sc01831a

**Published:** 2016-08-22

**Authors:** Thomas H. Pillow, Jack D. Sadowsky, Donglu Zhang, Shang-Fan Yu, Geoffrey Del Rosario, Keyang Xu, Jintang He, Sunil Bhakta, Rachana Ohri, Katherine R. Kozak, Edward Ha, Jagath R. Junutula, John A. Flygare

**Affiliations:** a Research & Early Development , Genentech, Inc. , 1 DNA Way , South San Francisco , CA 94080 , USA . Email: thomashp@gene.com

## Abstract

Disulfide bonds provide a bioactivatable connection with applications in imaging and therapy. The circulation stability and intracellular release of disulfides are problematically coupled in that increasing stability causes a corresponding decrease in cleavage and payload release. However, an antibody offers the potential for a reversible stabilization. We examined this by attaching a small molecule directly to engineered cysteines in an antibody. At certain sites this unhindered disulfide was stable in circulation yet cellular internalization and antibody catabolism generated a disulfide catabolite that was rapidly reduced. We demonstrated that this stable connection and facile release is applicable to a variety of payloads. The ability to reversibly stabilize a labile functional group with an antibody may offer a way to improve targeted probes and therapeutics.

## Abstract 2

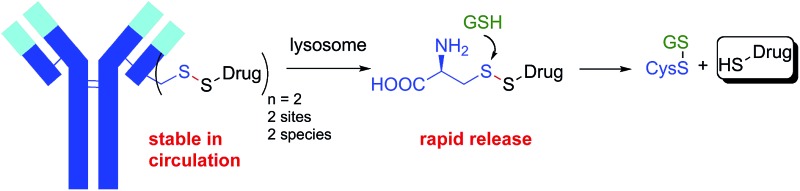
A novel bioconjugation strategy utilizes a disulfide bond to site-specifically couple a small molecule drug to an antibody, enabling both high circulation stability and quick intracellular release.

## Introduction

**Fig. 1:**
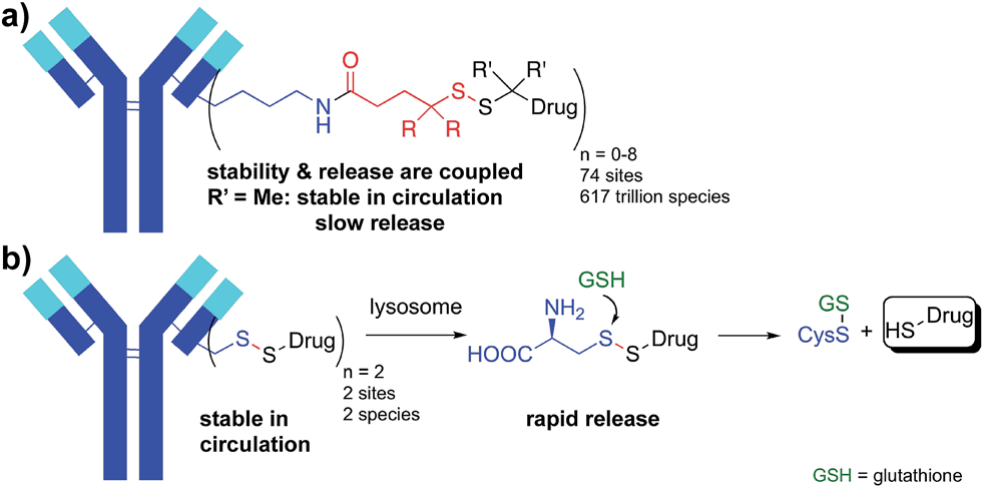
Antibody-small molecule drug conjugates with disulfide linkers. (a) Heterogeneous Lys conjugates formed through heterobifunctional cross-linkers have a stability and release that are coupled. (b) Decoupling stability and release: site-specific Cys conjugates effect drug release through lysosomal proteolysis and subsequent disulfide reduction.

Utilizing proteins or other polymers to deliver small molecule payloads *in vivo* is a well-recognized and validated strategy. Many applications have emerged that rely on this approach and these conjugated systems are able to dramatically change the half-life, solubility, and therapeutic index of the small molecule. One of the critical components in these conjugates is the connection between the small molecule and large molecule delivery vehicle. The disulfide bond is a bioactivatable connection that has been utilized for reversibly connecting protein toxins,^1^ chemotherapeutic drugs,^2–8^ and probes^9^ to carrier molecules such as antibodies for around 40 years.^10,11^ The majority of bioconjugates incorporating a disulfide connection have introduced the disulfide *via* a heterobifunctional crosslinker between the drug or probe and lysine (Lys) residues on the protein. Given the number of reactive Lys residues in most proteins, the result is a highly heterogeneous conjugate with a varying number of drugs at a large number of sites (Fig. 1a).^12^ This heterogeneity presents challenges for characterization and analysis, but more importantly can reduce activity and increase toxicity.^13,14^

The second even bigger challenge when attaching small molecules to proteins through heterobifunctional disulfide linkers is that the stability of the conjugate is coupled to the ability to release the payload. In many applications, including antibody-drug conjugates (ADCs), high stability of the disulfide in circulation and low stability of the disulfide within the target cell are desired. Disulfides are reduced in the cytosol of cells where the concentration of reduced glutathione (GSH) is 1–10 mM whereas cysteine (Cys) is the most abundant reactive thiol in plasma with concentrations between 8–11 µM.^15^ While this 1000 fold difference in reactive thiol concentration is the basis for the use of disulfides in drug delivery, the lack of exquisite selectivity for reduction by GSH *versus* Cys leads to a coupling of stability and release. Unhindered disulfides provide the most facile reduction in the cytosol but the lowest stability in circulation. Increasing the stability of the disulfide bond with adjacent alkyl groups increases circulation half-life, but hinders release of the free drug in the target cell. Achieving the desired stability and release is therefore a balancing act. The disulfide conjugates that have advanced into human clinic trials have typically had an intermediate level of alkyl substitution around the disulfide (1-2 methyl groups on either side of the disulfide).^16,17^ For example, in maytansine disulfide conjugates (Fig. 1a, R = H, R′ = Me; SPDB-DM4), the drug becomes deconjugated from the antibody in circulation with a half-life of ∼9 days^18^ while a major catabolite in tumors for the first 4 days is the unreduced disulfide.^19^ While poorly reducible and even non-cleavable linkers for some payloads can afford efficacious ADCs, they are often limited to targets with high and homogeneous antigen expression.^20^ The approach of balancing stability and release has led to a sacrifice in one or both potentially critical attributes.

Described herein is an approach to simultaneously achieve high stability in circulation and fast payload release in a target cell for disulfide-linked antibody conjugates. To achieve high stability in circulation we connected a small molecule drug directly *via* a disulfide to the two free thiols of a Cys-engineered antibody at a variety of mutant sites. Target cell internalization and degradation of the antibody by the lysosome then permitted facile cleavage of the disulfide bond by cytosolic reductants (*e.g.* GSH) and release of the drug (Fig. 1b). Our approach has several advantages. Rather than extending the disulfide away from the protein as is done with most disulfide conjugates through the use of heterobifunctional linkers, the direct approach brings the disulfide bond as close as possible to the antibody. By connecting the disulfide directly to the antibody we can take advantage of a steric protection resulting from reduced solvent accessibility at some sites in a three-dimensional folded protein. While others have investigated attaching drugs through disulfide bonds at different sites in smaller proteins, none of these approaches resulted in *in vivo* stability sufficient for antibody-mediated delivery and stability remained problematically coupled to release.^21–23^ This approach also reduces the heterogeneity in the resulting conjugate since drugs are specifically attached to the engineered Cys residues. Since only a single bond links the drug to the antibody, cleavage releases unmodified drug and antibody fragment without the need for a linker.^24^

## Results and discussion

**Scheme 1:**
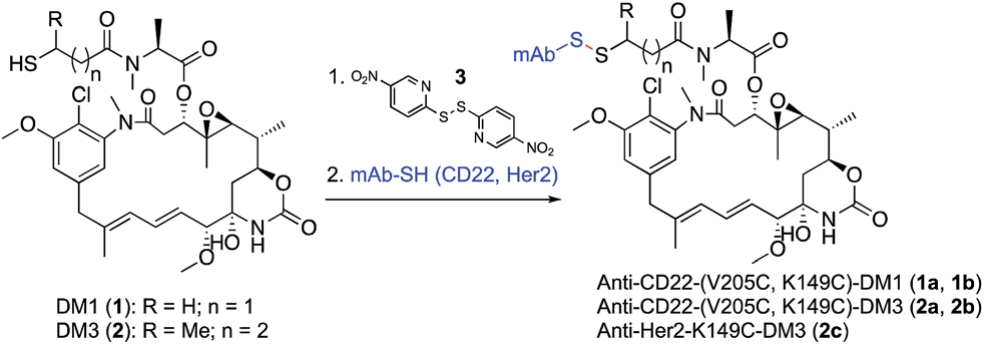
Synthesis of site-specific antibody-maytansine conjugates through disulfide activation. Drug-to-antibody ratios (DARs) were uniform, ranging between 1.8 and 2.0.

To test the design concept we utilized thiol-containing derivatives of the natural product maytansine.^25^ Derivatives DM1 (**1**) and DM3 (**2**), differing primarily in the addition of a methyl group next to the disulfide, were first treated with activating agent **3** followed by reaction with the engineered Cys of an anti-CD22 antibody (Scheme 1). We selected antibody site LC-V205C as a starting point as it offered the greatest stability for maleimide-linked conjugates in previous studies.^26^

**Fig. 2:**
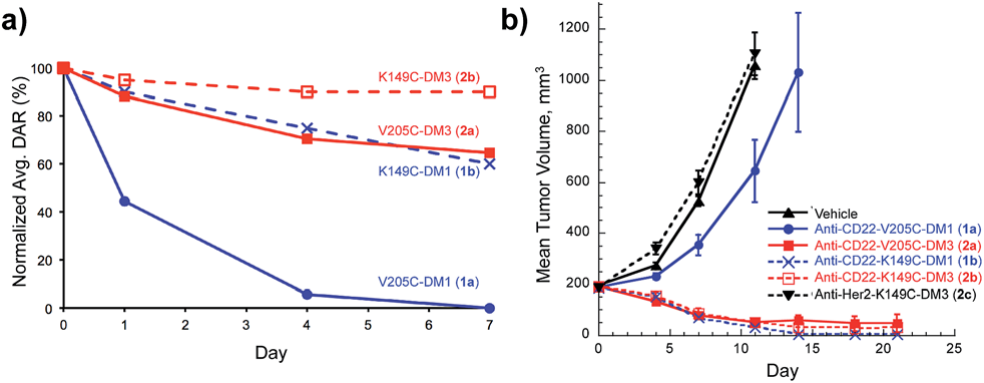
*In vivo* stability and efficacy of site-specific antibody-maytansine disulfide conjugates. (a) *In vivo* stability of antibody maytansine disulfide conjugates in mice. SCID mice were dosed intravenously with 3 mg kg^–1^ of anti-CD22-DM1 and DM3 (V205C and K149C). At the indicated time points, blood was drawn for determination of the average DAR normalized to day 0 using an affinity-capture LC-MS method.^27^ (b) *In vivo* efficacy of antibody maytansine disulfide conjugates in mice bearing BJAB.luc human non-Hodgkin lymphoma xenografts. SCID mice were subcutaneously implanted with 20 million tumor cells and administered a single IV dose (day 0) of vehicle or conjugates at 3 mg kg^–1^ (∼90 µg m^–2^) when average tumor size reached ∼190 mm^3^. Mean tumor volumes (±SEM) are plotted over time (days post dose).

We evaluated the V205C-DM1 conjugate for *in vivo* stability (Fig. 2a). Interestingly, while we previously demonstrated that conjugates linked to V205C using maleimide chemistry were highly stable with minimal drug loss *in vivo* up to 21 days, we found that a DM1 conjugate (**1a**) linked at the same site through a disulfide bond was quite unstable.

Within one day, about half of the unhindered drug (DM1, **1**) was lost in circulation. Mass spectrometry analysis indicated that a disulfide displacement had occurred, resulting in loss of drug and addition of Cys and GSH to the antibody (Fig. S1†).

Based on these results we sought antibody sites that would generate more stable disulfide conjugates. A screen of several mutation sites led ultimately to the identification of LC-K149C as a stable conjugation site for disulfides (manuscript in preparation). When we attached DM1 (**1**) through a disulfide to K149C (**1b**), only about 10% of the drug was lost after one day, and even after seven days, more than 50% of the drug remained attached (*versus* 56% and 100% loss after 1 and 7 days, respectively, for LC-V205C, **1a**). The stability of unsubstituted disulfide conjugate **1b** is comparable to that of the Lys-linked disulfide conjugate with two neighboring methyl groups (SPDB-DM4).^18^ Furthermore, addition of just one methyl group next to the disulfide (DM3, **2**) resulted in conjugates possessing increased stability at both sites on the antibody with the LC-K149C conjugate (**2b**) losing only 10% of the drug after seven days.

We next evaluated anti-tumor effects of anti-CD22 disulfide conjugates in a human lymphoma tumor xenograft in mice (Fig. 2b). Conjugates were administered at a single dose of 3 mg kg^–1^ and tumor volume was measured over time. Consistent with the *in vivo* stability data, the least stable conjugate, V205C-DM1 (**1a**), was also the least efficacious, resulting in modest tumor growth delay. Increasing stability either through site (K149C-DM1, **1b**) or addition of a methyl group (V205C-DM3, **2a**) resulted in complete tumor regression. Furthermore, this activity was target mediated as anti-Her2-K149C-DM3 (**2c**), the non-target control conjugate, showed no detectable activity and was equivalent to the vehicle control.

**Fig. 3:**
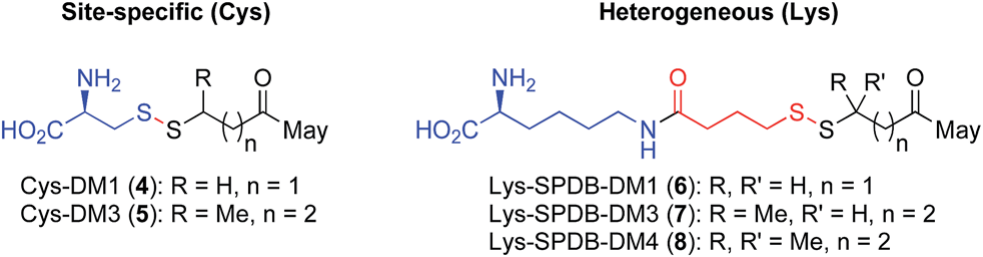
Maytansine disulfide conjugate lysosomal catabolites: Cys catabolites expected from site-specific disulfide conjugates and Lys catabolites resulting from heterogeneous conjugates prepared with heterobifunctional linkers.

Having demonstrated that relatively high stability of the disulfide in circulation can be achieved by appropriate choice of conjugation site, we evaluated the effectiveness of drug release in a target cell. It is well established that both Lys- and Cys-linked antibody-drug conjugates are completely catabolized in the lysosome to an amino acid-linker-drug species, wherein the amino acid portion is a remnant of the antibody attachment site.^19,20,28^ For Lys linked antibody conjugates the catabolites observed are Lys-SPDB-DM1 (**6**), -DM3 (**7**), and -DM4 (**8**) (Fig. 3).^19,20^ For our site-specific Cys disulfide conjugates, the expected catabolites are Cys-DM1 (**4**) and Cys-DM3 (**5**). Based on previous data showing that endosomes/lysosomes are oxidizing,^9^ we posit that disulfide metabolites escape the lysosome and are reduced in the cytosol. Thus to compare release of maytansinoid payloads in a target cell we measured stability of the Lys- and Cys-linked catabolites in the presence of a 3.3 fold stoichiometric excess of reductants DTT or GSH (Table 1).

**Table 1:** Reduction of Cys and Lys disulfide catabolites using dithiothreitol (DTT) and glutathione (GSH)

Metabolite	Site-specific	# Me grps^*a*^	% remaining^*b*^ (DTT, 15 m)	% remaining^*c*^ (GSH, 24 h)
Cys-DM1 (**4**)	Yes	0	67	16
Lys-SPDB-DM1 (**6**)	No	0	87	62
Cys-DM3 (**5**)	Yes	1	97	73
Lys-SPDB-DM3 (**7**)	No	1	98	98
Lys-SPDB-DM4 (**8**)	No	2	100	99

^*a*^Number of methyl groups on adjacent atom to disulfide.

^*b*^15 µM metabolite, 50 µM DTT.

^*c*^15 µM metabolite, 50 µM GSH.

The number of methyl groups on the carbon atom adjacent to the disulfide was the primary determinant for the stability of the disulfide bond, with an increasing number resulting in a sterically encumbered disulfide more resistant to reduction.^29^ Interestingly, within disulfides containing the same substitution, the Cys-catabolites were more readily reduced than Lys-catabolites. This trend is likely a result of the reduced p*K*_a_ for the thiol in Cys, making it a better leaving group than the alkyl thiols present in the Lys-modified metabolites. These results, as well as those at higher glutathione concentrations (Table S1†), suggest that drug release inside a target cell is more facile for Cys-linked conjugates than for Lys-linked conjugates.

**Fig. 4:**
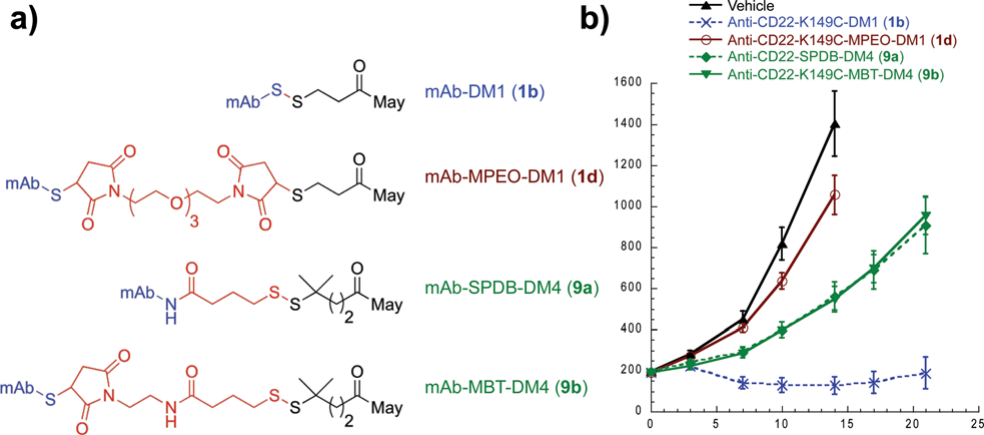
Impact of release on efficacy for antibody-maytansine conjugates. (a) Structures of antibody-maytansine conjugates. (b) *In vivo* efficacy of antibody maytansine disulfide conjugates in mice bearing BJAB.luc human non-Hodgkin lymphoma xenografts. SCID mice were subcutaneously implanted with 20 million tumor cells and administered a single IV dose (day 0) of vehicle or conjugates at 50 µg m^–2^ (drug dose) when average tumor size reached ∼190 mm^3^. Mean tumor volumes (±SEM) are plotted over time (days post dose).

With an understanding of the impact of disulfide stability on efficacy and disulfide substitution on chemical reduction, we set out to evaluate the influence of disulfide release on efficacy (Fig. 4). We selected the human lymphoma tumor xenograft BJAB, as this model discriminates between conjugates containing cleavable and non-cleavable linkers.^30^ Anti-CD22 conjugates were administered at a single maytansine dose of 50 µg m^–2^ to allow comparison between Lys- and Cys-linked conjugates with differential drug loads. Confirming the importance of release, the non-cleavable maleimide control MPEO-DM1 (**1d**) was completely stable at this new site (Fig. S2†) yet showed minimal activity. Significantly, while the disulfide conjugate K149C-DM1 (**1b**, Cys-linked) has similar circulation stability to SPDB-DM4 (**9a**, Lys-linked), it possessed superior efficacy, indicating that the ability to decouple stability from release results in improved activity *in vivo*. To confirm that this was not a result of antibody site (Cys *vs*. Lys) or drug loading, we made MBT-DM4 (**9b**), a Cys-linked conjugate with the same hindered disulfide as SPDB-DM4 (9a), and demonstrated that the two conjugates were equally efficacious.

**Fig. 5:**
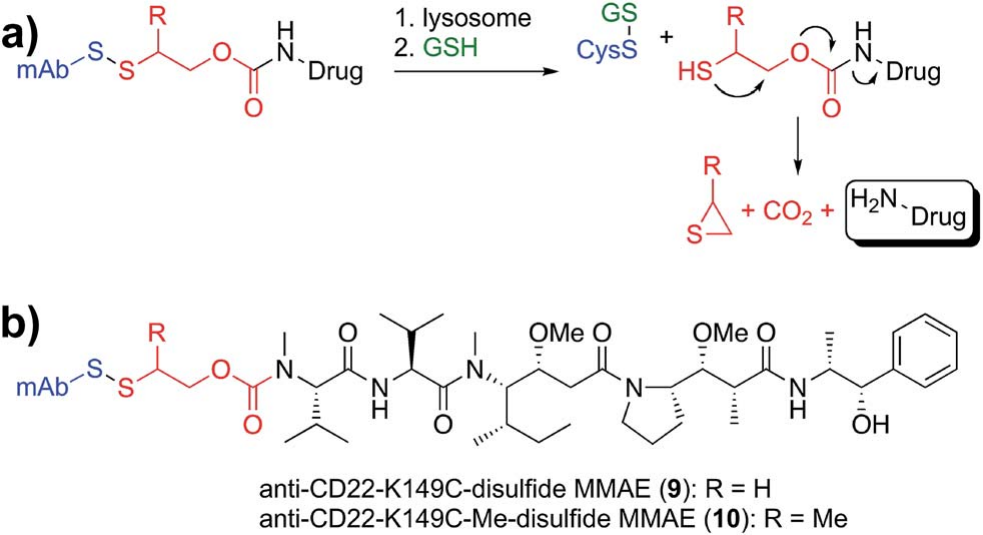
Application of site-specific disulfide conjugates to amine-containing drugs. (a) The disulfide conjugate is degraded by proteolysis in the lysosome and reduced by glutathione to generate a free thiol that cyclizes to generate thiirane, carbon dioxide, and the amine-containing drug. (b) Disulfide MMAE antibody conjugates were generated with (**10**) and without (**9**) a neighboring methyl group.

Lastly, we sought to develop Cys-linked disulfide chemistry that would enable traceless release of non-thiol drugs (*e.g.*, amines), while retaining high stability in circulation and rapid release of payload (Fig. 5). Traceless, self-immolative disulfide linkers have been used to release phosphates,^31^ alcohols,^32–34^ hydrazides,^35^ and amines^36,37^ for a variety of imaging and therapeutic applications.^11^ Immolation for some of these has been proposed to occur through either cyclization to a 3-membered ring (thiirane or episulfide) or cyclization to a 5-membered cyclic thiocarbonate.^38–40^ We sought to evaluate the immolative disulfide linker for release of amines, a chemical functional group exemplified by many small molecule drugs and probes. We found that treatment of an immolative disulfide linker-drug (100 µM) with a physiologically relevant concentration of a reductant (Cys, 2 mM) resulted in the production of free drug and thiirane (Fig. S3†) as demonstrated by LC-MS.

Since immolating disulfides have not previously been connected to an antibody we wanted to establish whether stability was maintained in circulation. One point of concern is the fact that the electron withdrawing nature of the carbamate group lowers the p*K*_a_ of the thiol and therefore makes it a better leaving group relative to the thiol in the maytansine disulfides described above. Nevertheless, we discovered that conjugates of the two immolating disulfides (**9**, **10**, Fig. 5b) had an *in vivo* stability profile similar to that of the similarly substituted maytansines described above (Fig. S4†). Furthermore, the stability of disulfides at site K149C is not antibody specific as ADCs with anti-Her2 have the same stability as those with anti-CD22 (Fig. S5†).

## Conclusions

In summary, we have demonstrated for the first time, the decoupling of stability and release in a disulfide antibody conjugate. This decoupling was accomplished by establishing conjugation chemistry in conjunction with antibody engineering that allowed antibody-mediated stabilization of the disulfide while in circulation and rapid deconjugation upon complete antibody catabolism inside a target cell. Our site-specific conjugates are as or more stable than disulfide conjugates employing heterobifunctional crosslinkers but generate catabolites that more readily release the payload. Lastly, we extended our work to immolating disulfide linkers that combine the advantages of antibody protection, rapid cleavage inside a target cell and release of amine-containing drugs. In addition to providing stabilization to disulfides, we believe that the approach of utilizing an antibody to protect a labile chemical functional group will allow improvement in the targeting, half-life, and metabolic stability of small molecule drugs and probes for therapeutic, diagnostic and imaging applications.
