# Supplementary material for: Decoupling stability and release in disulfide bonds with antibody-small molecule conjugates
Source: Chem Sci. 2016 Aug 22;8(1):366–70. doi: 10.1039/c6sc01831a (PMC5365059; doi:10.1039/c6sc01831a)

Table of Contents

1. **Figures S1-S5 and Table S1 (pg. 2-5)**
2. **Reagents and Methods (pg. 6-8)**
3. **Disulfide Conjugation Reactions and Efficiencies (pg. 9-10)**
4. **Experimental Procedures (pg. 11-20)**
5. **^{1}H NMR Spectra (pg. 21-28)**

2. Figures S1-S5 and Table S1

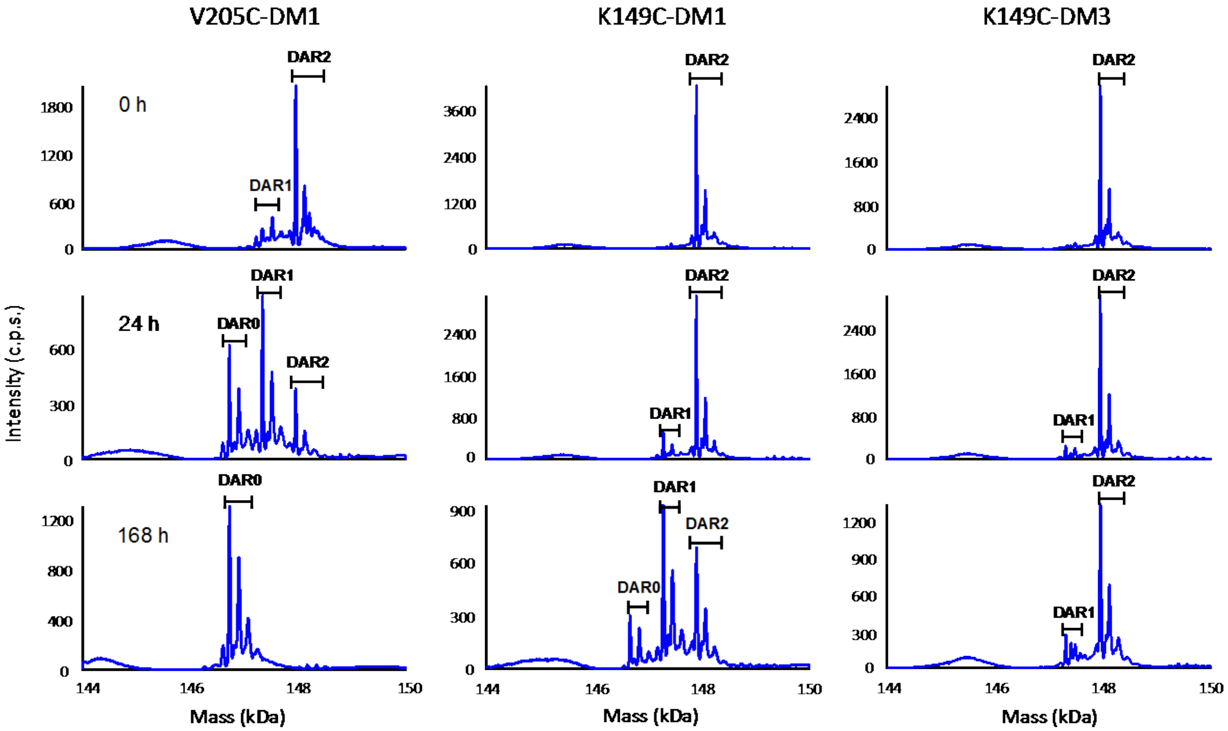

Figure S1. Mass spectra of *in vivo* mouse stability samples for antibody-maytansine disulfide conjugates. LC-MS indicates that loss of drug for anti-CD22-V205C-DM1 (**1a**), anti-CD22-K149C-DM1 (**1b**), and anti-CD22-K149C-DM3 (**2b**) is effected through displacement with Cys or GSH as observed by loss of drug MW and additions of 120 Da (Cys) or 306 (GSH). This loss can be reduced by changing the antibody site (V205C to K149C) as well as by addition of a methyl group (DM1 to DM3).

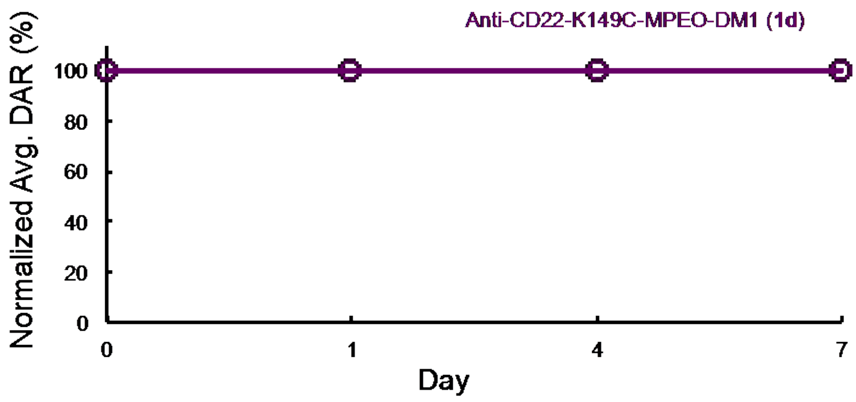

Figure S2. *In vivo* mouse stability of maleimides at site K149C. LC/MS was used to observe the stability of anti-CD22 MPEO-DM1 (**1d**) in mice over 7 days. Despite containing two maleimides, no drug loss was observed for this ADC for 7 days.

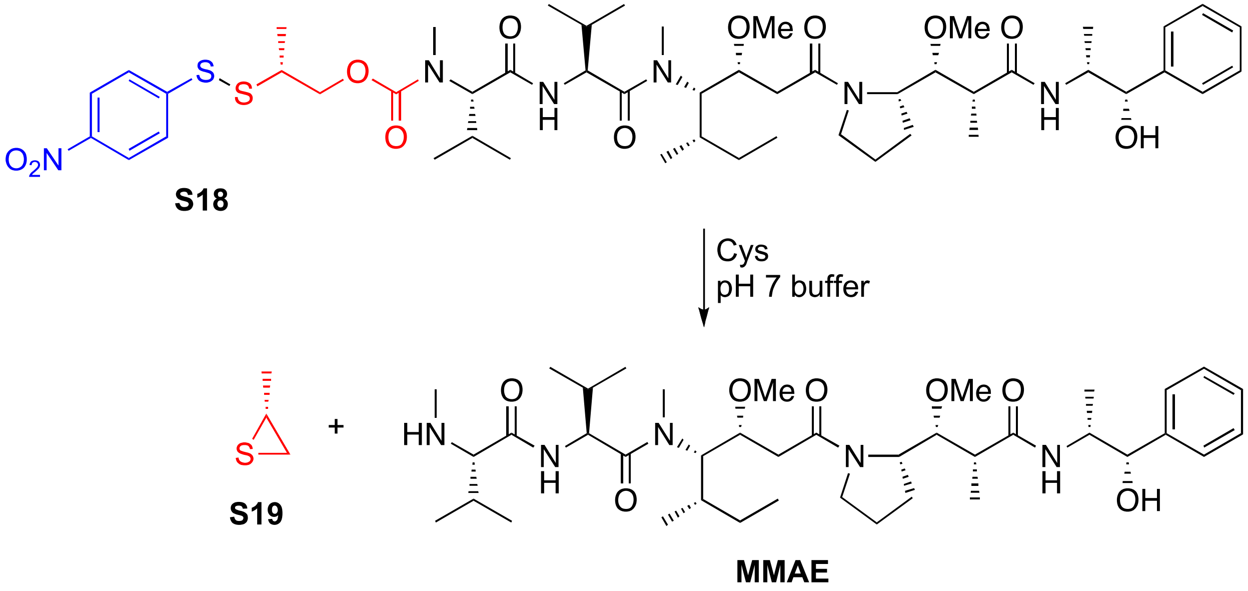

Figure S3. Probing mechanism of release for immolative disulfide linker-drugs. Activated disulfide **S18**, was used as a starting material to support immolation of free thiols generated by the reduction of disulfide metabolites formed in cells. **S18** (100 μM) was reduced with Cys (2 mM) in a pH 7 buffer and the byproducts were analyzed by LC-MS. Methyl thiirane (**S19**) and MMAE were observed within 2 hours of reduction.

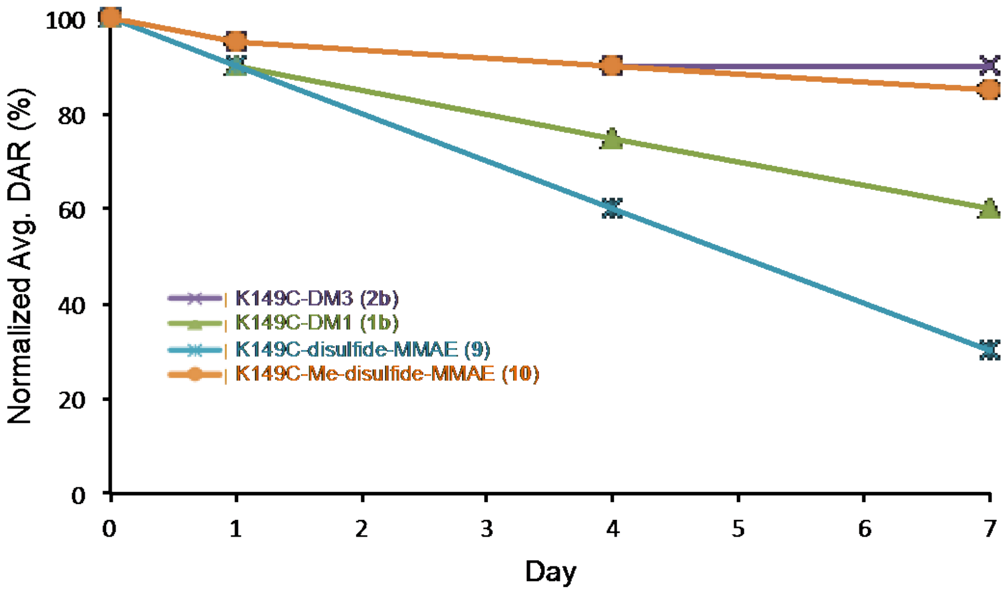

Figure S4. *In vivo* mouse stability of disulfide-linked MMAE and maytansine antibody conjugates. The immolating disulfide-linked MMAE ADCs anti-CD22-K149C-disulfide MMAE (**9**) and anti-CD22-K149C-Me-disulfide-MMAE (**10**) displayed a similar *in vivo* stability profile to that of similarly substituted disulfide-linked maytansine ADCs anti-CD22-K149C-DM1 (**1b**) and anti-CD22-K149C-DM3 (**2b**). The stability of both MMAE and maytansine conjugates with unhindered disulfides (**9** and **1b** respectively) were significantly improved by addition of one methyl group next to the disulfide (**10** and **2b**).

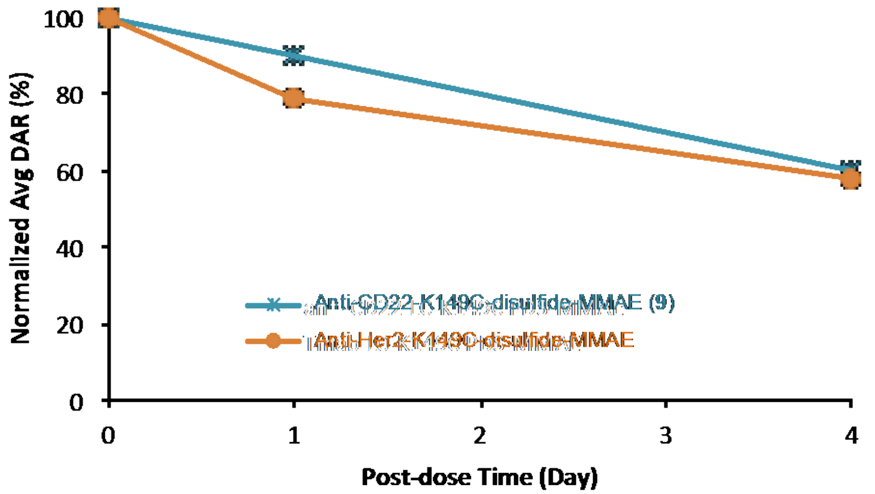

Figure S5. *In vivo* mouse stability of anti-CD22 and anti-Her2 disulfide-linked MMAE antibody conjugates. The *in vivo* stability of disulfide-MMAE linker-drug (**S11**) conjugated at K149C on two different antibodies was identical, supporting the antibody-independent nature of disulfide stability.

Table S1. Reduction of cysteine and lysine disulfide catabolites using glutathione (GSH).

Metabolite	Site-specific	# Me grps[a]	% Remaining (GSH, 1 h)[b]
cys-DM1 (**4**)	Yes	0	0.15
lys-SPDB-DM4 (**8**)	No	2	27

[a] Number of methyl groups on adjacent atom to disulfide. [b] 15 μM metabolite and 4 □M GSH to give a 267 fold stoichiometric excess of reductant.

2. Reagents and Methods

Preparation of conjugates. In general, conjugates were prepared by reacting the two engineered Cys residues on THIOMABs with activated disulfide analogs of the maytansine or MMAE linker-drugs. Antibodies were produced in CHO cells and purified using standard methods. In these antibodies, the engineered Cys residues are present as mixed disulfides with cysteine or glutathione, which must be removed ("deblocked") to enable conjugation to the engineered Cys sulfhydryl groups. To accomplish this, the antibodies were partially reduced with DTT, purified, reoxidized with dehydroascorbic acid (DHAA) and purified again into a succinate buffer (10 mM succinate, pH 5.0, 150 mM NaCl, 2 mM EDTA) to give the deblocked THIOMAB. The pH of the deblocked antibody was adjusted with 1M Tris, pH 8.5 (75 mM final Tris concentration). To the pH-adjusted THIOMAB solution was added 3 equivalents of nitropyridyl disulfide maytansine or MMAE linker-drugs (10 mM stock in DMF) suitably diluted to give 10% DMF in the final reaction solution. The conjugation reactions were allowed to proceed at room temperature until completion as indicated by LC-MS analysis of the reaction mixture (3-4 hours). Reaction mixtures were diluted 5-fold into 20 mM histidine-acetate, pH 5.5 buffer and loaded onto S maxi strong-cation exchange columns (Pierce), washed several times with histidine-acetate and eluted in 20 mM histidine-acetate, pH 5.5, 300 mM NaCl. Conjugates were formulated into 20 mM histidine-acetate, pH 5.5, 240 mM sucrose by dialysis. Conjugates were analyzed for drug-to-antibody ratio (DAR) by reverse-phase LC-MS (PLRP-S column, Agilent TOF instrument), for aggregation by analytical SEC (Shodex column, Agilent HPLC instrument) and for endotoxin by LAL assay (Charles River instrument). All conjugates had DAR values of 1.8 - 2.0, were > 98.5% monomeric and had endotoxin levels < 0.1 EU/mg.

Reduction assay. Compounds at 15 µM were incubated with either 50 µM DTT or 50 µM GSH (3.3 fold stoichiometric excess of reductant, or 4 mM GSH (267 fold stoichiometric excess of reductant) in 100 mM Tris buffer pH 7.0 containing 5% methanol at 37 °C. Aliquots were taken at specified time points and the samples were analyzed by LC-MS on Sciex TripleTOF 5600 on a Hypersil Gold C18 column (100 x 2.1, 1.9 µM, Thermo Scientific). The column was eluted by a gradient of buffer A (0.1% formic acid in 10 mM ammonium acetate) to buffer B (0.1% formic acid in 10 mM ammonium acetate in 90% acetonitrile), 5% to 25% B in 8 min, to 75% B in 5 min, and to 95% B in 1 min.

Identification of thiirane (9) immolation byproduct. For analysis of the thiirane **S19** byproduct, 100 µM of the disulfide **S18** was incubated with 2 mM cysteine in 100 mM

Tris buffer pH 7.0 containing 5% methanol for 2 h at 37 °C. Methyl thiirane **S19** was detected (M/Z found: 75.0282, calculated: 75.0268) which co-eluted with a synthetic standard at a retention time of 2.61 min.

Animal studies. All animal studies were carried out in compliance with the National Institutes of Health guidelines for the care and use of laboratory animals and were approved by the Institutional Animal Care and Use Committee at Genentech, Inc. The efficacy of anti-CD22 drug conjugates was evaluated in a mouse xenograft model of BJAB.luc human non-Hodgkin lymphoma. The BJAB.luc cell line was obtained from the Genentech cell line repository. Cells were maintained in RPMI 1640 supplemented with 10% FBS (Sigma) and 1% L-glutamine. This cell line was authenticated by short tandem repeat (STR) profiling using the Promega PowerPlex 16 System and compared with external STR profiles of cell lines to determine cell line ancestry. To establish a subcutaneous xenograft model, the tumor cells (20 million cells in 0.2mL Hank's Balanced Salt Solution; Hyclone) were inoculated subcutaneously into the flanks of female CB17 SCID mice (Charles Rivers Laboratories). When mean tumor size reached the desired volume, the mice were divided into groups of 8 mice with similar mean tumor size and received a single intravenous injection of antibody drug conjugate through the tail vein. The results were plotted as mean tumor volume ± SEM of each group over time. Blood samples were collected via retro-orbital bleeds and used to derive plasma for stability analysis by affinity capture LC-MS.

Affinity capture LC-MS. To determine the *in vivo* stability of ADCs, affinity capture LC-MS was performed as described previously (*16*, *27*). Briefly, human CD22 extracellular domain (ECD) was biotinylated and immobilized onto streptavidin-coated paramagnetic beads (Invitrogen) in a 96-well plate, and then the ECD-bead system was used to capture anti-CD22 disulfide conjugates by incubating with approximately 40 µL of mouse plasma samples for 2 h at room temperature. The captured ADCs were then washed with HBS-EP buffer (10 mM Hepes [pH 7.4], 150 mM NaCl, 3.4 mM ethylenediaminetetraacetic acid [EDTA], 0.005% Surfactant P20) (GE Healthcare) and deglycosylated using N-Glycanase (Prozyme) at 37 °C overnight. After extensive washing of the beads with HBS-EP, water and 10% acetonitrile, the ADC analytes were eluted using 30% acetonitrile in water with 1% formic acid. A KingFisher 96 magnetic particle processor (Thermo Electron) was used to mix, wash, gather, and transfer the paramagnetic beads in the above steps. A volume of 10 µL of the eluents was analyzed by LC-MS using a TripleTOF 5600 mass spectrometer (AB Sciex). Chromatographic separation of ADCs was performed on a nanoACQUITY UPLC® system (Waters Corporation) equipped with a PS-DVB monolithic column (500 um i.d. X 5 cm) (Thermo

Scientific). Raw data was deconvoluted using Analyst TF 1.6 software, and the average drug-to-antibody ratio was calculated based on the peak areas of different DAR species (DAR0-DAR2).

Materials. All solvents and reagents were used as obtained from commercial sources. HRMS was determined with a Dionex LC Ultimate3000 and ThermoScientific Q Exactive orbitrap mass spectrometer. Samples were prepared in a mixture of MeOH/acetonitrile/water (4:4:1 v/v/v) at a concentration of 200 μM for high resolution LC-MS analysis. Samples of 5 μL were injected into the LC-MS system and were analyzed by a 10 min gradient on HPLC and HRMS with electrospray ionization. Bruker instruments (400 and 500 MHz) were used for NMR characterization. Chemical shifts are expressed as δ units using tetramethylsilane as the internal standard. Purifications were done using a Waters HPLC with a Gemini-NX 10u C18, 100 x 30 mm column under formic acid (acid) or ammonium formate (base) modified conditions.

3. Disulfide Conjugation Reactions and Efficiencies

Preparation of conjugates is described above. Values for Drug to antibody ratio (DAR) is placed outside of parenthesis and conversion is calculated based on a DAR 2 being 100% conversion.

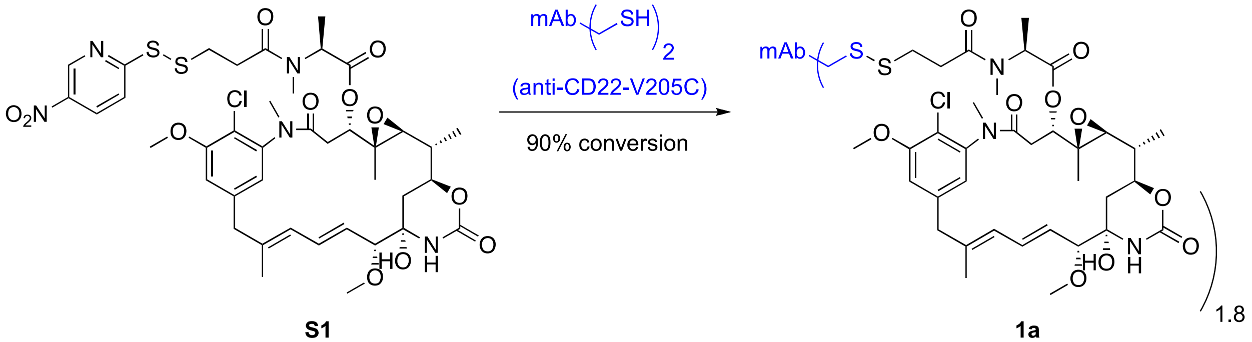

S1 1a

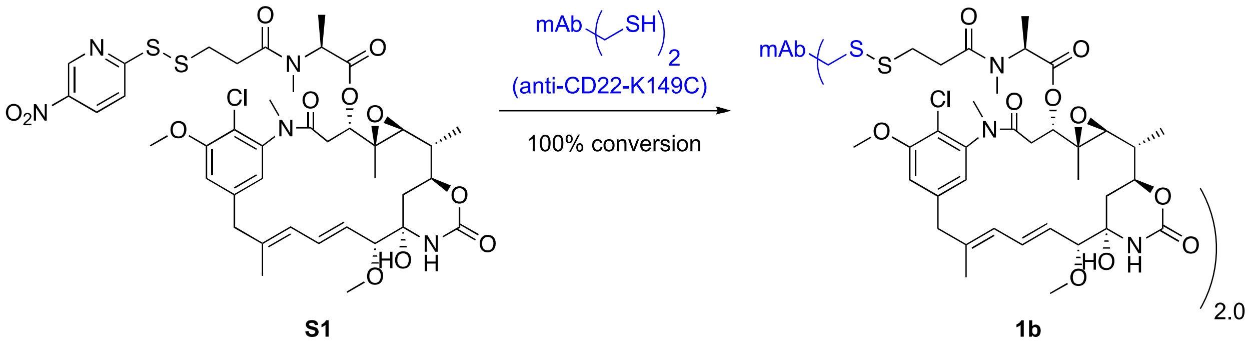

S1 1b

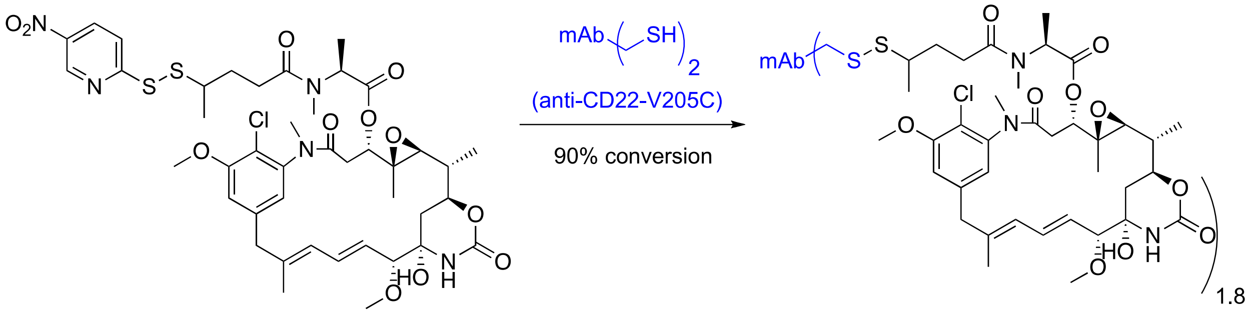

2a

S2

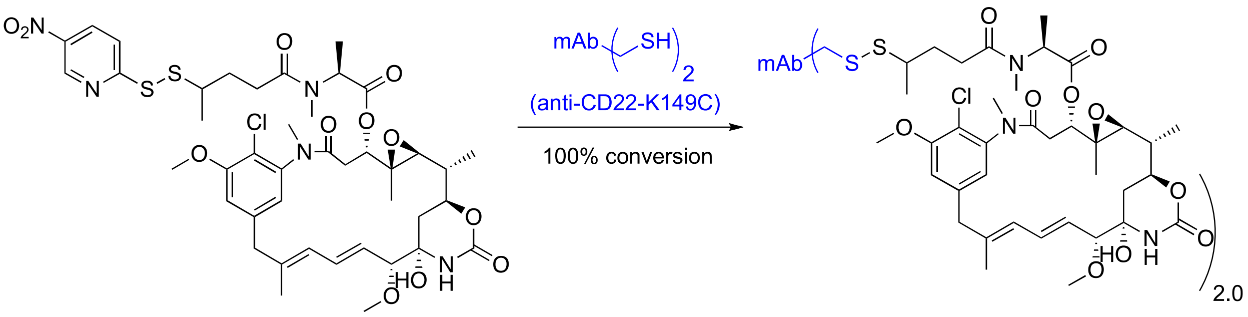

S2 2b

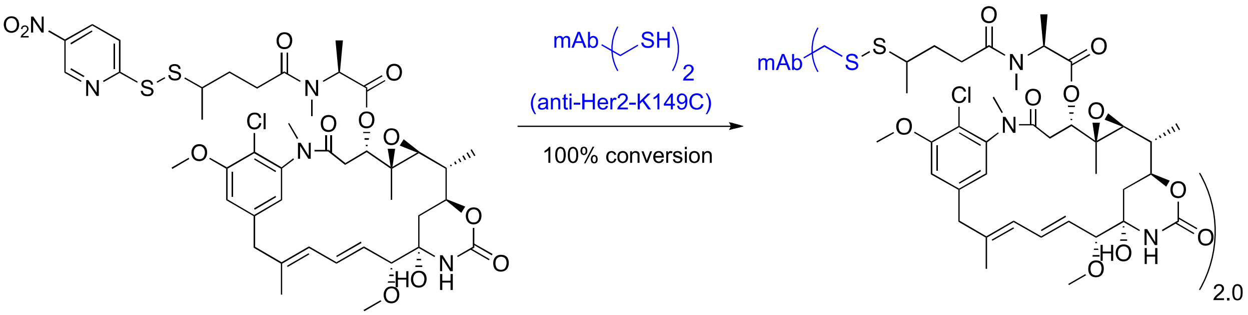

S2 2b

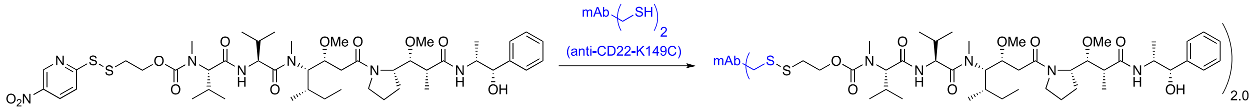

S11 9

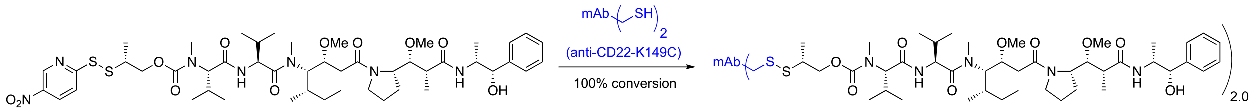

S18 10

4. Experimental Procedures

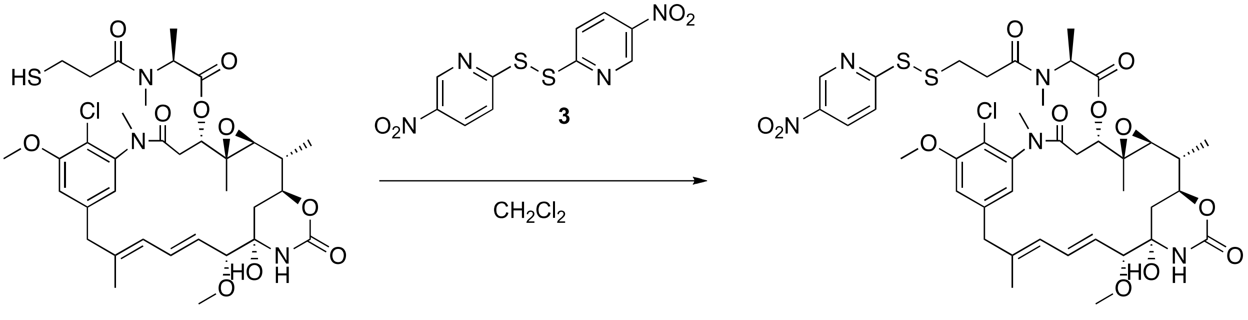

DM1 (**1**) **S1**

To a solution of 2,2'-bis(5-nitropyridyl) disulfide (**3**) (67.3 mg, 0.22 mmol) in CH_2Cl_2 (5.0 mL) was added DM1 (**1**) (80.0 mg, 0.11 mmol). The mixture was stirred at 40 °C for 1 h, concentrated, and purified by prep-TLC (15% MeOH in CH_2Cl_2, Rf = 0.5) to give **S1** (80.0 mg, 0.11 mmol) as a white solid. ^{1}H NMR (400 MHz, DMSO-*d*$_6$) δ 9.09 (d, *J* = 2.7 Hz, 1H), 8.44 (dd, *J* = 8.9, 2.7 Hz, 1H), 7.87 (d, *J* = 8.9 Hz, 1H), 7.00 (d, *J* = 1.8 Hz, 1H), 6.87 (s, 1H), 6.53 (m, 2H), 6.36 (d, *J* = 1.8 Hz, 1H), 5.92 (d, *J* = 1.3 Hz, 1H), 5.62 – 5.49 (m, 1H), 5.29 (q, *J* = 6.7 Hz, 1H), 4.51 (dd, *J* = 12.0, 2.8 Hz, 1H), 4.14 – 3.99 (m, 1H), 3.87 (s, 3H), 3.48 (d, *J* = 9.0 Hz, 1H), 3.25 (s, 3H), 3.21 – 3.08 (m, 1H), 3.08 (s, 3H), 3.07 – 2.87 (m, 3H), 2.78 (d, *J* = 9.7 Hz, 1H), 2.70 (s, 3H), 2.68 – 2.62 (m, 1H), 2.42 (dd, *J* = 14.5, 12.0 Hz, 1H), 2.00 (dd, *J* = 14.5, 2.8 Hz, 1H), 1.54 (s, 3H), 1.50 – 1.38 (m, 2H), 1.27 – 1.19 (m, 2H), 1.17 (d, *J* = 6.8 Hz, 3H), 1.11 (d, *J* = 6.3 Hz, 3H), 0.74 (s, 3H). ^{13}C NMR (101 MHz, DMSO) δ 170.9, 170.4, 168.6, 167.1, 155.6, 151.7, 145.2, 142.5, 141.5, 141.4, 138.6, 133.0, 132.9, 129.0, 125.6, 121.6, 119.6, 117.5, 114.1, 88.6, 80.4, 78.1, 73.6, 67.2, 60.4, 56.9, 56.6, 52.1, 45.8, 38.1, 36.8, 35.5, 33.7, 33.4, 32.3, 30.1, 15.4, 14.9, 13.5, 11.9. HRMS (ESI): m/z [M + H] calcd. for $C_{40}H_{51}ClN_5O_{12}S_2$: 892.2664 Found: 892.2648.

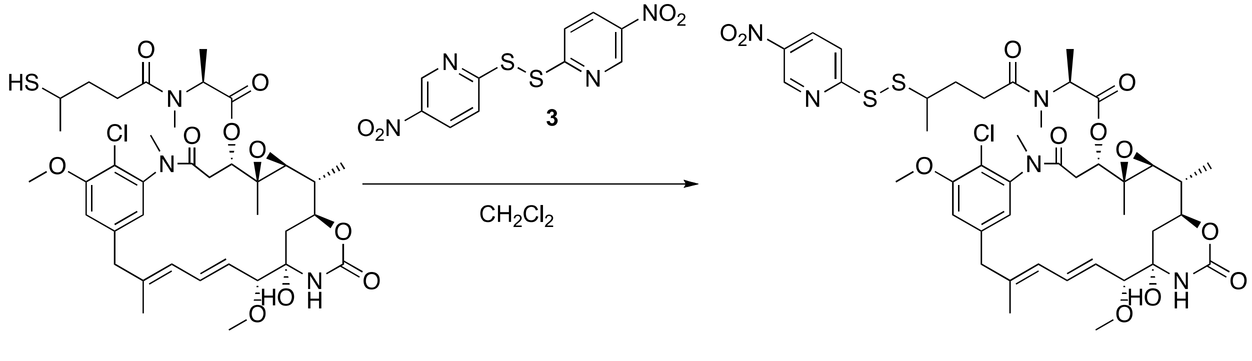

DM3 (**2**) **S2**

To a solution of 2,2'-bis(5-nitropyridyl) disulfide (**3**) (15.0 mg, 0.020 mmol) in CH_2Cl_2 (2.0 mL) was added DM3 (**2**) (18.2 mg, 0.060 mmol), and the mixture was stirred at 18 °C for 72 h. The mixture was purified by prep-TLC (10% MeOH in CH_2Cl_2, Rf = 0.5) to give **S2** (3.0 mg, 0.0032 mmol, 16.5% yield) as a white solid. ^{1}H NMR (400 MHz, DMSO-d_6, 2 diastereomers) δ 9.29 – 9.13 (m, 2H), 8.60 – 8.45 (m, 2H), 8.02 – 7.90 (m, 2H), 7.25 – 7.14 (m, 2H), 6.87 (s, 2H), 6.61 – 6.48 (m, 6H), 5.91 (s, 2H), 5.60 – 5.48 (m, 2H), 5.36 – 5.26 (m, 2H), 4.58 – 4.47 (m, 2H), 4.12 – 4.01 (m, 2H), 3.94 (s, 6H), 3.54 – 3.44 (m, 2H), 3.26 (s, 6H), 3.22 – 3.13 (m, 4H), 3.11 (s, 3H), 3.07 (s, 3H), 2.83 – 2.75 (m, 2H), 2.72 (s, 3H), 2.71 (s, 3H), 2.69 – 2.52 (m, 2H), 2.42 – 2.24 (m, 2H), 2.09 – 1.89 (m, 4H), 1.88 – 1.76 (m, 2H), 1.59 (s, 3H), 1.58 (s, 3H), 1.51 – 1.40 (m, 6H), 1.34 – 1.25 (m, 2H), 1.19 (d, J = 6.7 Hz, 6H), 1.15 (d, J = 6.9 Hz, 6H), 1.14 – 1.10 (m, 6H), 0.91 – 0.81 (m, 2H), 0.78 (s, 3H), 0.77 (s, 3H). ^{13}C NMR (101 MHz, DMSO, 2 diastereomers) δ 171.6, 171.5, 171.1, 168.6, 168.1, 155.8, 151.7, 145.19, 145.15, 142.6, 141.7, 141.6, 138.7, 133.0, 132.93, 132.89, 130.1, 129.0, 125.7, 122.0, 121.9, 120.1, 117.7, 117.6, 114.4, 88.6, 80.4, 78.1, 73.6, 67.2, 60.5, 57.0, 56.6, 52.0, 46.3, 46.2, 45.9, 38.2, 36.8, 35.74, 35.69, 32.4, 31.7, 31.2, 31.0, 30.8, 30.1, 29.54, 29.48, 29.3, 29.1, 29.0, 27.0, 25.6, 22.5, 20.5, 20.3, 15.5, 14.9, 14.4, 13.5, 11.8. HRMS (ESI): m/z [M + H] calcd. for $C_{42}H_{55}ClN_5O_{12}S_2$: 920.2977 Found: 920.3029.

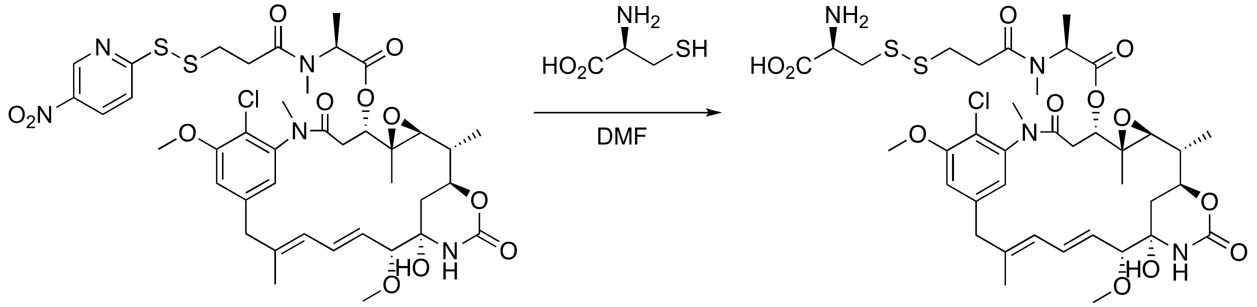

S1 **4**

To a solution of **S1** (80.0 mg, 0.09 mmol) in DMF (2.0 mL) was added cysteine (108.6 mg, 0.90 mmol). The mixture was stirred at 6 °C for 10 min. The mixture was purified by prep-HPLC (basic) to give **4** (22 mg, 0.0254 mmol, 28.3% yield) as a white solid. ^{1}H NMR (400 MHz, DMSO-d_6) δ 7.16 (d, J = 1.9 Hz, 1H), 6.88 (s, 1H), 6.70 – 6.43 (m, 3H), 5.92 (s, 1H), 5.64 – 5.50 (m, 1H), 5.30 (q, J = 6.8 Hz, 1H), 4.53 (dd, J = 11.9, 2.9 Hz, 1H), 4.16 – 4.00 (m, 1H), 3.93 (s, 3H), 3.98 – 3.88 (m, 1H), 3.53 – 3.40 (m, 4H), 3.26 (s, 3H), 3.31 – 3.21 (m, 2H), 3.22 – 3.04 (m, 2H), 3.09 (s, 3H), 2.84 – 2.73 (m, 2H), 2.69 (s, 3H), 2.77 – 2.57 (m, 1H), 2.57 – 2.43 (m, 1H), 2.09 – 1.95 (m, 1H), 1.59 (s, 3H), 1.52 – 1.39 (m, 3H), 1.25 (d, J = 12.2 Hz, 2H), 1.14 (dd, J = 18.2, 6.6 Hz, 6H), 0.78 (s, 3H). ^{13}C NMR (101 MHz, DMSO) δ 171.0, 170.8, 168.6, 168.3, 155.7, 151.7, 141.8, 141.6, 138.7, 133.0, 129.0, 125.7, 122.0, 117.5, 114.5, 88.6, 80.5, 78.2, 73.6, 67.3, 60.5, 57.0,

56.6, 55.4, 53.1, 52.1, 46.0, 38.2, 36.8, 35.8, 33.5, 32.6, 32.4, 30.2, 15.5, 14.9, 13.5, 11.9. HRMS (ESI): m/z [M + H] calcd. for $C_{38}H_{54}ClN_4O_{12}S_2$: 857.2868 Found: 857.2850.

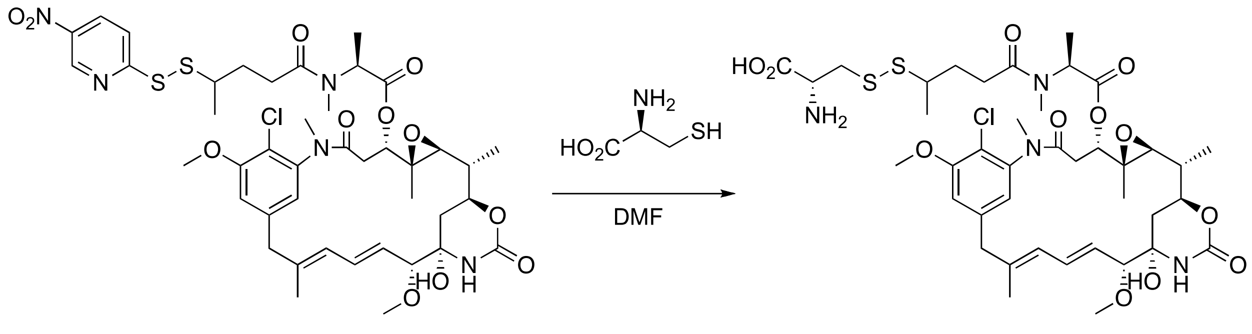

S2 **5**

To a solution of **S2** (18.0 mg, 0.020 mmol) in DMF (3.0 mL) was added cysteine (23.69 mg, 0.20 mmol). The mixture was stirred at 6 °C for 10 min and was purified by prep-HPLC (basic) to give the product **5** (5.0 mg, 0.0056 mmol, 28.5% yield) as a white solid. ^{1}H NMR (500 MHz, DMSO-d_6, 2 diastereomers) δ 7.26 – 7.12 (m, 2H), 6.92 (s, 2H), 6.66 – 6.50 (m, 6H), 5.94 (s, 2H), 5.66 – 5.46 (m, 2H), 5.46 – 5.23 (m, 2H), 4.63 – 4.39 (m, 2H), 4.17 – 3.99 (m, 2H), 3.95 – 3.89 (m, 8H), 3.53 – 3.40 (m, 8H), 3.25 (s, 6H), 3.23 – 3.16 (m, 2H), 3.13 – 3.06 (m, 6H), 2.84 – 2.75 (m, 2H), 2.76 – 2.63 (m, 6H), 2.58 – 2.52 (m, 2H), 2.49 – 2.35 (m, 2H), 2.31 – 2.18 (m, 2H), 2.09 – 2.00 (m, 2H), 1.85 – 1.64 (m, 2H), 1.59 (s, 6H), 1.51 – 1.40 (m, 4H), 1.28 – 1.21 (m, 4H), 1.22 – 1.18 (m, 2H), 1.18 – 1.14 (m, 8H), 1.14 – 1.11 (m, 8H), 1.11 – 1.08 (m, 2H), 1.06 – 1.03 (m, 2H), 1.00 – 0.94 (m, 2H), 0.89 – 0.81 (m, 2H), 0.80 – 0.76 (m, 6H). ^{13}C NMR (126 MHz, DMSO, 2 diastereomers) δ 171.74, 171.67, 171.1, 168.6, 155.7, 151.7, 141.7, 141.6, 138.7, 133.0, 129.0, 125.7, 122.0, 117.6, 114.3, 88.6, 80.4, 78.2, 73.6, 67.3, 60.5, 57.0, 56.6, 52.0, 45.9, 38.2, 36.8, 36.4, 35.7, 35.6, 32.4, 31.4, 30.9, 30.7, 30.1, 29.9, 21.2, 21.1, 20.4, 20.2, 20.1, 15.5, 14.9, 14.4, 13.5, 11.8. HRMS (ESI): m/z [M + H] calcd. for $C_{40}H_{58}ClN_4O_{12}S_2$: 885.3181 Found: 885.3228.

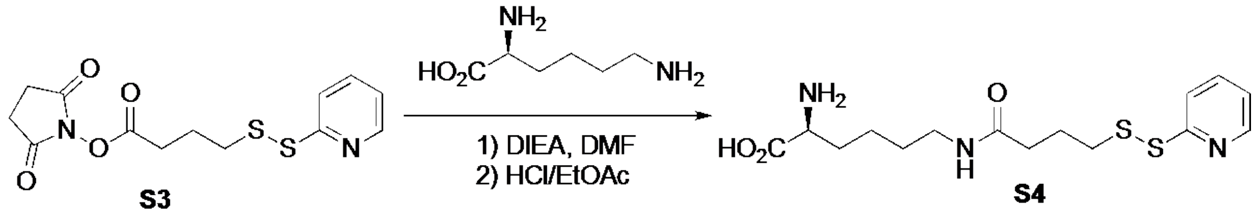

S3 **S4**

A solution of *N*-succinimidyl 4-(2-pyridyldithio)butanoate (SPDB, **S3)** (200.0 mg, 0.61 mmol), lysine (226 mg, 0.92 mmol), and DIEA (158 mg, 1.23 mmol) in DMF (8.0 mL)

was stirred at 18 °C for 18 h. The mixture was concentrated and an HCl solution in EtOAc (6.0 mL, 24 mmol) was added to the mixture. It was stirred at 18 °C for 3 h, and concentrated. The resulting residue was purified by prep-HPLC to give the desired product **S4** (100 mg, 0.28 mmol, 45% yield) as a white solid. ^{1}H NMR (400 MHz, D2O/DMSO-d_6) δ 8.45 (d, J = 4.8 Hz, 1H), 7.80 (dd, J = 22.6, 7.6 Hz, 3H), 7.24 (t, J = 6.1 Hz, 1H), 3.09 – 3.04 (m, 1H), 3.02 – 2.94 (m, 2H), 2.83 (t, J = 7.2 Hz, 2H), 2.18 (t, J = 7.3 Hz, 2H), 1.90 – 1.78 (m, 2H), 1.77 – 1.62 (m, 1H), 1.61 – 1.45 (m, 1H), 1.42 – 1.19 (m, 4H). HRMS (ESI): m/z [M + H] calcd. for $C_{15}H_{24}N_3O_3S_2$: 358.1259 Found: 358.1269.

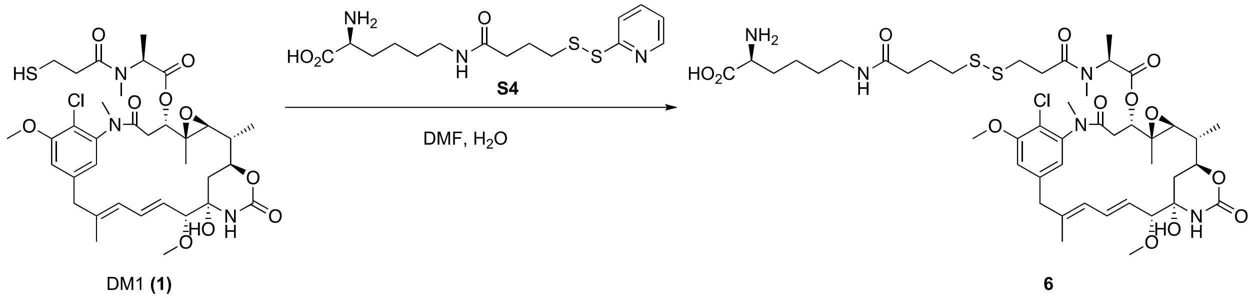

A solution of compound **S4** (21.8 mg, 0.060 mmol) and DM1 (**1**) (30.0 mg, 0.040 mmol) in DMF (3.0 mL) /water (3.0 mL) was stirred at for 40 °C for 6 h. The mixture was purified by prep-HPLC (basic) to give the desired product **6** (15.4 mg, 0.0155 mmol, 38.1% yield) as a white solid. ^{1}H NMR (400 MHz, DMSO-d_6) δ 7.82 (t, J = 5.5 Hz, 1H), 7.18 (d, J = 1.8 Hz, 1H), 6.89 (s, 1H), 6.64 – 6.49 (m, 3H), 5.56 (dd, J = 14.1, 9.1 Hz, 1H), 5.37 – 5.27 (m, 1H), 4.53 (dd, J = 12.0, 2.8 Hz, 1H), 4.20 – 3.99 (m, 1H), 3.93 (s, 3H), 3.54 – 3.38 (m, 3H), 3.25 (s, 3H), 3.25 – 3.17 (m, 2H), 3.15 – 3.02 (m, 1H), 3.11 (s, 3H), 3.05 – 2.95 (m, 2H), 2.94 – 2.76 (m, 4H), 2.72 (s, 3H), 2.72 – 2.51 (m, 2H), 2.48 (d, J = 7.3 Hz, 1H), 2.12 – 2.00 (m, 3H), 1.78 – 1.60 (m, 3H), 1.59 (s, 3H), 1.58 – 1.51 (m, 1H), 1.46 (td, J = 11.6, 4.7 Hz, 2H), 1.41 – 1.21 (m, 8H), 1.18 (d, J = 6.8 Hz, 3H), 1.12 (d, J = 6.4 Hz, 3H), 0.78 (s, 3H). ^{13}C NMR (101 MHz, DMSO) δ 171.4, 171.0, 170.8, 170.2, 168.6, 155.7, 151.7, 141.8, 141.7, 138.8, 133.0, 129.0, 125.7, 122.1, 117.6, 114.4, 88.7, 80.5, 78.2, 73.6, 67.2, 60.5, 57.0, 56.6, 54.6, 52.2, 45.9, 38.8, 38.2, 37.3, 36.8, 35.7, 34.2, 33.7, 33.5, 32.4, 31.2, 30.2, 29.4, 25.1, 23.1, 15.5, 14.9, 13.5, 11.8. HRMS (ESI): m/z [M + H] calcd. for $C_{45}H_{67}ClN_5O_{13}S_2$: 984.3865 Found: 984.3841.

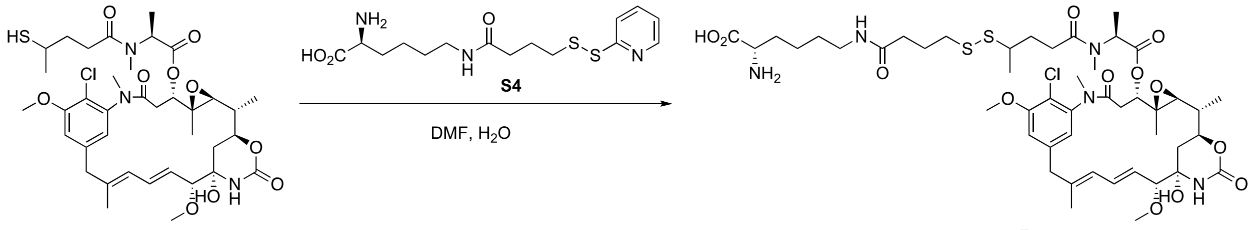

DM3 (**2**)

7

A mixture of DM3 (**2**) (20.0 mg, 0.030 mmol) and compound **S4** (14.0 mg, 0.040 mmol) in DMF (4.0 mL)/ water (2.0 mL) was stirred at for 40 °C for 6 h. The resulting residue was purified by prep-HPLC (basic) to give desired product **7** (10.9 mg, 0.0107 mmol, 40.8% yield) as a white solid. ^{1}H NMR (400 MHz, DMSO-d_6) δ 7.82 (td, *J* = 5.6, 2.4 Hz, 1H), 7.19 (s, 1H), 6.90 (s, 1H), 6.66 – 6.52 (m, 3H), 5.57 (ddd, *J* = 14.7, 9.0, 3.2 Hz, 1H), 5.42 – 5.27 (m, 1H), 4.53 (dd, *J* = 12.1, 2.8 Hz, 1H), 4.17 – 4.00 (m, 1H), 3.93 (s, 3H), 3.52 – 3.40 (m, 3H), 3.25 (s, 3H), 3.24 – 3.15 (m, 2H), 3.11 (s, 3H), 3.11 – 3.07 (m, 1H), 3.07 – 2.95 (m, 2H), 2.96 – 2.76 (m, 2H), 2.71 (d, *J* = 1.4 Hz, 3H), 2.63 – 2.51 (m, 4H), 2.30 (dt, *J* = 16.0, 7.1 Hz, 1H), 2.16 – 1.99 (m, 3H), 1.99 – 1.86 (m, 1H), 1.83 – 1.62 (m, 5H), 1.59 (s, 3H), 1.59 – 1.40 (m, 3H), 1.40 – 1.22 (m, 6H), 1.22 – 1.07 (m, 9H), 0.78 (s, 3H).^{13}C NMR (101 MHz, DMSO) δ 171.9, 171.8, 171.5, 171.1, 170.2, 168.6, 155.7, 151.7, 141.8, 141.7, 141.6, 138.7, 133.0, 129.0, 125.7, 122.1, 117.6, 114.4, 88.7, 80.5, 78.1, 73.6, 67.3, 60.5, 57.0, 56.6, 54.6, 52.0, 52.0, 45.9, 45.4, 44.9, 38.83, 38.78, 38.2, 36.8, 35.7, 34.2, 32.4, 31.3, 31.2, 30.9, 30.3, 30.2, 29.4, 25.3, 25.2, 23.1, 21.2, 20.7, 15.5, 14.9, 13.5, 11.8. HRMS (ESI): m/z [M + H] calcd. for $C_{47}H_{71}ClN_5O_{13}S_2$: 1012.4178 Found: 1012.4172.

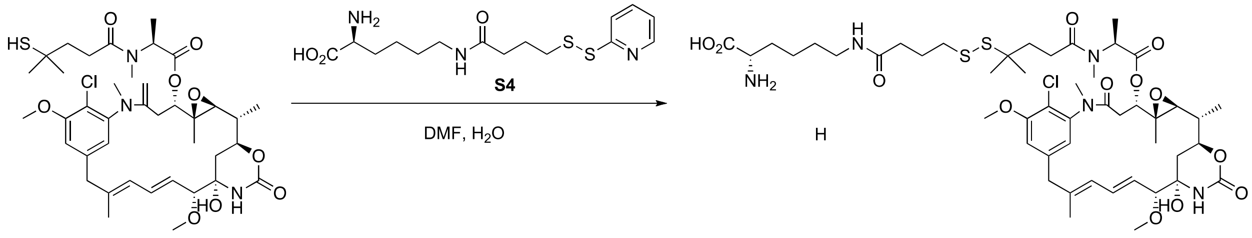

DM4 (**S5**)

8

A mixture of DM4 (**12**) (15.0 mg, 0.020 mmol) and compound **S4** (13.7 mg, 0.040 mmol) in DMF (3.0 mL) / Water (3.0 mL) was stirred at 40 °C for 6 h. The residue was purified by prep-HPLC (basic) to give desired product **8** (11.1 mg, 0.0107 mmol, 55.7% yield) as a white solid. ^{1}H NMR (400 MHz, DMSO-d_6) δ 7.82 (t, *J* = 5.5 Hz, 1H), 7.18 (d, *J* = 1.8 Hz, 1H), 6.89 (s, 1H), 6.66 – 6.49 (m, 3H), 5.58 (dd, *J* = 14.7, 9.0 Hz, 1H), 5.38 – 5.28 (m, 1H), 4.53 (dd, *J* = 11.9, 2.9 Hz, 1H), 4.07 (t, *J* = 12.2, 10.4, 2.1 Hz, 1H), 3.93 (s, 3H), 3.52 – 3.41 (m, 4H), 3.25 (s, 3H), 3.30 – 3.15 (m, 2H), 3.11 (s, 3H), 3.11 – 3.04 (m, 1H), 3.04 – 2.95 (m, 2H), 2.80 (d, *J* = 9.7 Hz, 1H), 2.72 (s, 3H), 2.59 (t, *J* = 7.6 Hz, 2H),

2.55 – 2.41 (m, 1H), 2.23 (ddd, J = 16.3, 11.6, 4.5 Hz, 1H), 2.10 (t, J = 7.4 Hz, 2H), 2.04 (dd, J = 14.4, 2.8 Hz, 1H), 1.88 (ddd, J = 14.0, 11.7, 4.9 Hz, 1H), 1.78 – 1.64 (m, 5H), 1.59 (s, 3H), 1.59 – 1.40 (m, 3H), 1.41 – 1.20 (m, 6H), 1.20 – 1.09 (m, 12H), 0.78 (s, 3H).^{13}C NMR (101 MHz, DMSO) δ 171.8, 171.5, 171.2, 170.2, 168.6, 155.8, 151.7, 141.8, 141.7, 138.6, 133.0, 129.1, 125.8, 122.0, 117.6, 114.3, 88.7, 80.5, 78.1, 73.6, 67.2, 60.5, 57.0, 56.6, 54.6, 52.0, 50.6, 46.0, 38.8, 38.2, 36.8, 36.3, 35.7, 34.2, 32.4, 31.2, 30.2, 29.4, 29.0, 28.1, 27.3, 25.3, 23.1, 15.5, 14.9, 13.5, 11.9. HRMS (ESI): m/z [M + H] calcd. for $C_{48}H_{73}ClN_5O_{13}S_2$: 1026.4335 Found: 1026.4327.

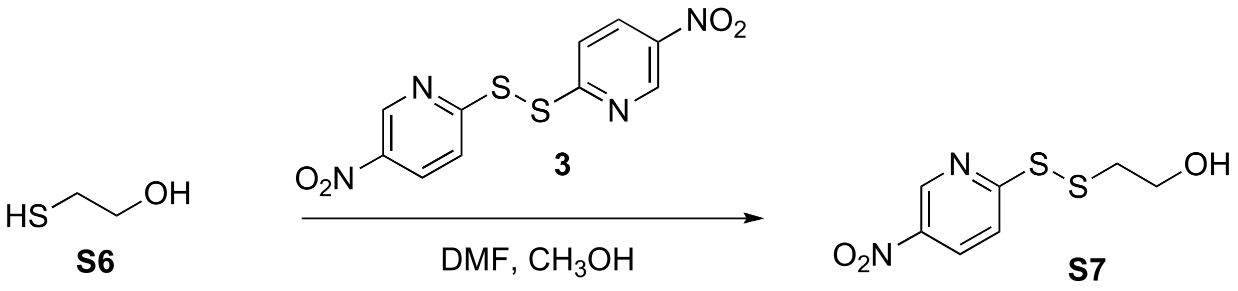

To a stirred solution of 2,2'-bis(5-nitropyridyl) disulfide (**3**) (3.0 g, 9.68 mmol) in MeOH/DMF (1:1, 10 mL), a solution of **S6** (380 mg, 4.84 mmol) in MeOH/DMF (1:1, 1.0 mL) was added dropwise over 5 min under N_2 at 15 °C. After the mixture was stirred at 15 °C for 16 h under N_2, it was concentrated and purified by prep-TLC (CH_2Cl_2/MeOH = 30/1) to give desired product **S7** (500 mg, 2.15 mmol, 44.4% yield). ^{1}H NMR (400 MHz, DMSO-d_6) δ 9.25 (d, J = 2.3 Hz, 1H), 8.59 (dd, J = 8.9, 2.8 Hz, 1H), 8.12 (d, J = 8.9 Hz, 1H), 5.00 (t, J = 5.4 Hz, 1H), 3.64 (dt, J = 6.0, 6.0 Hz, 2H), 2.99 (t, J = 6.2 Hz, 2H). ^{13}C NMR (101 MHz, DMSO) δ 168.2, 145.3, 142.7, 133.1, 120.0, 59.5, 41.8. HRMS (ESI): m/z [M + H] calcd. for $C_7H_9N_2O_3S_2$: 233.0055 Found: 233.0043.

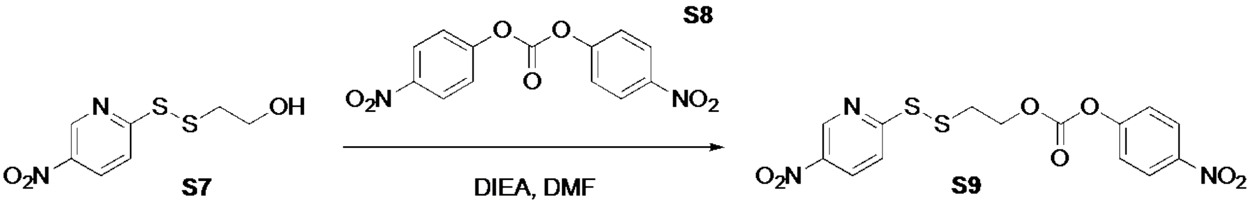

To a stirred solution of **S7** (50 mg, 0.215 mmol) and bis(4-nitrophenyl) carbonate (**S8**) (131 mg, 0.43 mmol) in DMF (3.0 mL) was added DIEA (56 mg, 0.43 mmol) at 15 ^{o}C. The mixture was stirred at 15 ^{o}C for 16 h under N_2. It was concentrated and purified by prep-TLC (CH_2Cl_2/MeOH = 30/1) to give desired product **S9** (60 mg, 0.15 mmol, 70%

yield). ^{1}H NMR (400 MHz, DMSO-d_6) δ 9.26 (d, *J* = 2.6 Hz, 1H), 8.59 (dd, *J* = 8.9, 2.7 Hz, 1H), 8.38 – 8.27 (m, 2H), 8.07 (d, *J* = 8.8 Hz, 1H), 7.60 – 7.51 (m, 2H), 4.50 (t, *J* = 5.9 Hz, 2H), 3.36 – 3.28 (m, 2H). ^{13}C NMR (101 MHz, DMSO) δ 167.2, 155.6, 152.2, 145.7, 145.4, 142.9, 133.1, 125.9, 123.0, 120.3, 67.1, 37.0. HRMS (ESI): m/z [M + H] calcd. for $C_{14}H_{11}N_3O_7S_2$: 397.0038 Found: 397.0130.

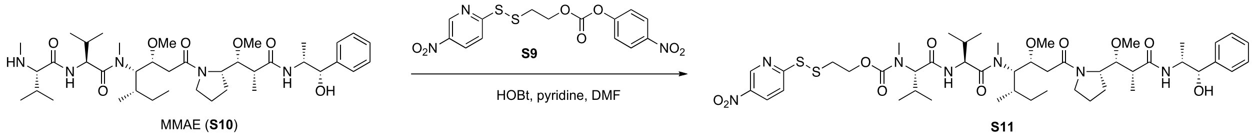

To a stirred solution of **S9** (30 mg, 0.0756 mmol), MMAE (**S10**) (49 mg, 0.068 mmol), and HOBt (2 mg, 0.015 mmol) in DMF (3 mL) was added pyridine (60 mg, 0.756 mmol) at 22 °C. The mixture was stirred at 20 °C for 16 h. It was purified by prep-HPLC (FA) to give the desired product **S11** (60 mg, 0.061 mmol, 81% yield). ^{1}H NMR (400 MHz, DMSO-d_6) δ 9.26 (d, *J* = 2.6 Hz, 1H), 8.66 – 8.49 (m, 1H), 8.30 – 7.98 (m, 2H), 7.74 (dd, *J* = 106.3, 8.6 Hz, 1H), 7.34 – 7.12 (m, 5H), 5.36 (dd, *J* = 26.2, 5.0 Hz, 1H), 4.80 – 4.36 (m, 3H), 4.34 – 4.12 (m, 3H), 4.09 – 3.89 (m, 3H), 3.82 – 3.38 (m, 3H), 3.25 – 3.22 (m, 3H), 3.21 – 3.15 (m, 4H), 3.14 – 2.94 (m, 4H), 2.91 – 2.74 (m, 4H), 2.41 (d, *J* = 16.0 Hz, 1H), 2.34 – 2.20 (m, 1H), 2.18 – 2.04 (m, 2H), 2.05 – 1.63 (m, 4H), 1.62 – 1.40 (m, 2H), 1.39 – 1.20 (m, 2H), 1.13 – 0.95 (m, 7H), 0.93 – 0.70 (m, 16H). ^{13}C NMR (126 MHz, DMSO) δ 172.8, 172.7, 170.1, 169.2, 167.5, 156.3, 155.9, 145.4, 144.1, 142.8, 133.1, 128.2, 128.2, 127.2, 127.1, 126.9, 126.8, 120.0, 85.8, 82.0, 78.1, 77.3, 75.2, 63.8, 63.2, 63.0, 61.4, 60.7, 59.1, 58.6, 57.6, 55.4, 54.6, 50.2, 49.6, 47.7, 46.7, 44.2, 43.6, 37.9, 37.6, 37.5, 35.5, 32.2, 32.0, 30.4, 30.2, 27.2, 25.8, 24.8, 23.6, 19.5, 19.4, 19.3, 19.1, 19.0, 18.8, 16.3, 16.0, 15.9, 15.8, 15.5, 10.9, 10.7. HRMS (ESI): m/z [M + H] calcd. for $C_{47}H_{74}N_7O_{11}S_2$: 976.4888 Found: 976.4946.

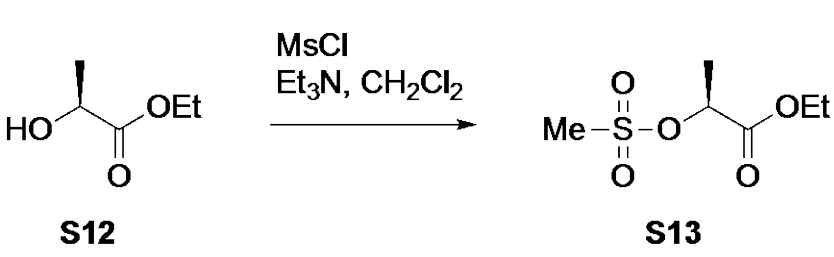

To a stirred solution of compound **S12** (17.1 g, 145 mmol) and Et_3N (43.9 g, 434 mmol) in CH_2Cl_2 (100 mL), methane sulfonyl chloride (MsCl) (33 g, 288 mmol) was added dropwise at 0 °C. After the reaction mixture was stirred at 23 °C for 2 h, it was extracted

with H_2O (80 mL x 2), washed with brine (80 mL), dried over Na_2SO_4 and concentrated to give **S13** (13.0 g) as a yellow oil which was carried on crude to the next step.

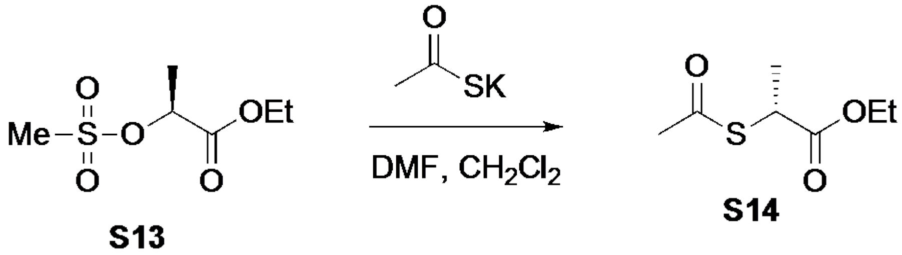

To a solution of **S13** (16.6 g, 84.65 mmol) in DMF/CH_2Cl_2 (50 mL/50 mL) was added potassium thioacetate (10.63 g, 93.08 mmol). After the mixture was stirred at 15 °C for 12 h, it was concentrated and the residue was dispersed in H_2O (450 mL), and extracted with MBTE (150 mL x 3). It was concentrated and purified by silica gel column (PE:EtOAc = 20:1) to give **S14** (9.0 g, 51 mmol, 28% over 2 steps) as a yellow oil. ^1H NMR (400 MHz, DMSO-d_6) δ 4.11 (p, J = 7.2 Hz, 3H), 2.35 (s, 3H), 1.40 (d, J = 7.3 Hz, 3H), 1.18 (t, J = 7.2 Hz, 3H). ^{13}C NMR (101 MHz, DMSO) δ 194.2, 171.6, 61.6, 40.9, 30.6, 17.9, 14.4. HRMS (ESI): m/z [M + H] calcd. for $C_7H_{13}O_3S$: 177.0585 Found: 177.0589.

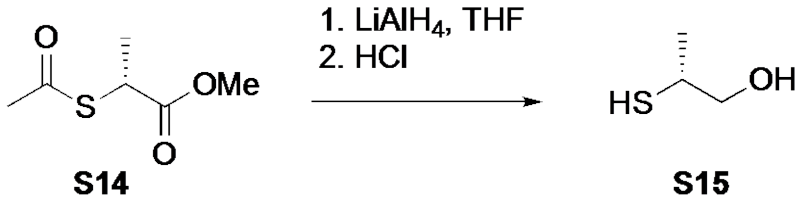

To a 500 mL round bottom flask was added $LiAlH_4$ (7.75 g, 204.20 mmol). Then THF (250 mL) was added at 0 °C. To the above suspension was added dropwise a solution of **S14** (10 g, 56.74 mmol) in THF (25 mL). After the mixture was stirred at reflux for 2 h, it was cooled to 0 °C. HCl solution (2 M, 100 mL) was added dropwise and the resultant mixture was stirred at16 °C for 1 h, then filtered through Celite, and washed with THF. The mixture was concentrated and extracted with CH_2Cl_2 (120 mL x 3). The combined organic layers were washed with brine (200 mL), dried over Na_2SO_4 and concentrated to give product **S15** (4.2 g, 46 mmol, 80% yield) as a yellow oil. ^1H NMR (400 MHz, DMSO-d_6) δ 4.88 (t, J = 5.6 Hz, 1H), 3.63 – 3.48 (m, 1H), 3.46 – 3.18 (m, 1H), 2.94 – 2.76 (m, 1H), 1.20 (d, J = 6.9 Hz, 3H). ^{13}C NMR (101 MHz, DMSO) δ 65.1, 48.6, 40.6, 40.4, 40.2, 40.0, 39.8, 39.6, 39.4, 17.5.

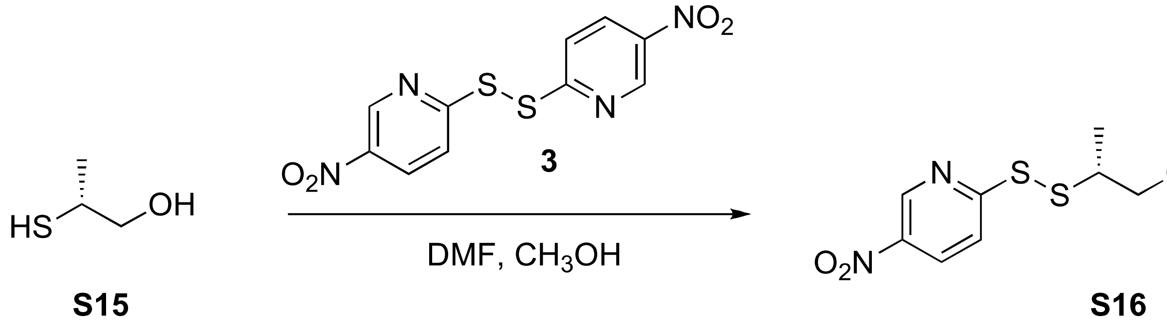

To a solution of 2,2'-bis(5-nitropyridyl) disulfide (**3**) (6.2 g, 20 mmol) in DMF (20 mL) and MeOH (35 mL) was added a solution of **S15** (0.9 g, 9.77 mmol) in MeOH (5 mL). After the mixture was stirred at 23 °C for 12 h, it was concentrated and dissolved in CH_2Cl_2 (200 mL). To the above solution was added MnO_2 (850 mg, 9.77 mmol), and stirred at 23 °C for 10 min and filtered. It was concentrated to give the crude product, which was purified by flash column chromatography (CH_2Cl_2) to give **S16** (900 mg, 3.65 mmol, 37.4% yield) as a yellow solid. It was further separated by SFC to give the optically pure product. ^{1}H NMR (400 MHz, DMSO-*d*$_6$) δ 9.23 (d, *J* = 2.4 Hz, 1H), 8.58 (dd, *J* = 8.9, 2.6 Hz, 1H), 8.12 (d, *J* = 9.1 Hz, 1H), 5.08 (t, *J* = 5.5 Hz, 1H), 3.57 – 3.42 (m, 2H), 3.20 – 3.10 (m, 1H), 1.26 (d, *J* = 6.9 Hz, 3H). ^{13}C NMR (101 MHz, DMSO) δ 168.7, 145.2, 142.7, 133.1, 120.0, 64.5, 49.2, 17.2. HRMS (ESI): m/z [M + H] calcd. for $C_8H_{11}N_2O_3S_2$: 247.0211 Found: 247.0231.

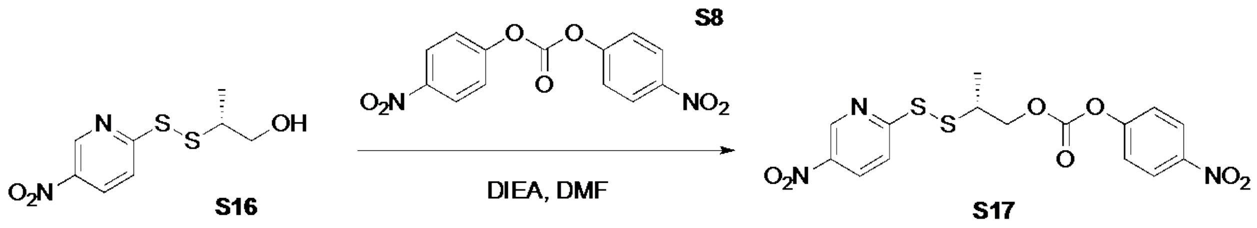

To a stirred solution of compound **S16** (50 mg, 0.203 mmol) and bis(4-nitrophenyl) carbonate (**S8**) (123 mg, 0.406 mmol) in DMF (2.0 mL) was added DIEA (78 mg, 0.609 mmol). The reaction mixture was stirred at 23 °C for 2 h. Solvent was removed under reduced pressure, and the residue was added to H_2O (40 mL) and extracted with CH_2Cl_2 (50 mL x 2). The organic phases were combined, dried, concentrated, and purified by prep-HPLC (PE/EtOAc = 3/1) to give compound **S17** (60 mg, 0.15 mmol, 72% yield) as a yellow solid. ^{1}H NMR (400 MHz, DMSO-*d*$_6$) δ 9.24 (d, *J* = 2.7 Hz, 1H), 8.58 (dd, *J* = 8.9, 2.6 Hz, 1H), 8.36 – 8.27 (m, 2H), 8.08 (d, *J* = 9.0 Hz, 1H), 7.60 – 7.52 (m, 2H), 4.40 – 4.35 (m, 2H), 3.64 – 3.51 (m, 1H), 1.36 (d, *J* = 6.9 Hz, 3H). ^{13}C NMR (101 MHz, DMSO) δ 167.5, 155.6, 152.2, 145.3, 142.9, 133.1, 125.9, 123.0, 120.3,

71.0, 44.8, 17.0. HRMS (ESI): m/z [M + H] calcd. for $C_{15}H_{14}N_3O_7S_2$: 412.0273 Found: 412.0261.

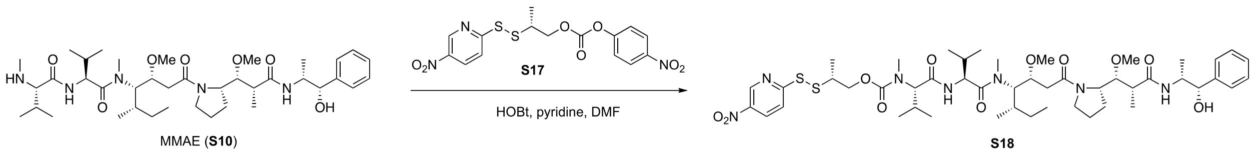

To a stirred solution of MMAE (**S10**) (30 mg, 0.042 mmol) and compound **S17** (34 mg, 0.084 mmol) in dry DMF (2.0 mL) was added HOBT (1.0 mg, 0.0042 mmol) and pyridine (33 mg, 0.42 mmol). The mixture was stirred at 23 °C for 12 h and then purified by prep-HPLC (acid) to give compound **S18** (23 mg, 0.023 mmol, 55% yield) as a white solid. ^{1}H NMR (400 MHz, DMSO-d_6) δ 9.24 (d, J = 2.7 Hz, 1H), 8.58 – 8.53 (m, 1H), 8.34 – 7.96 (m, 2H), 7.74 (dd, J = 106.7, 8.5 Hz, 1H), 7.34 – 7.11 (m, 5H), 5.36 (dd, J = 25.8, 5.0 Hz, 1H), 4.81 – 4.34 (m, 3H), 4.27 – 4.11 (m, 3H), 3.97 (dt, J = 21.4, 8.3 Hz, 3H), 3.86 – 3.39 (m, 3H), 3.28 (s, 1H), 3.24 (d, J = 7.2 Hz, 3H), 3.18 (d, J = 8.7 Hz, 3H), 3.15 – 2.95 (m, 4H), 2.94 – 2.77 (m, 4H), 2.41 (d, J = 16.0 Hz, 1H), 2.27 (dd, J = 16.0, 9.2 Hz, 1H), 2.17 – 2.06 (m, 2H), 2.05 – 1.63 (m, 4H), 1.62 – 1.40 (m, 2H), 1.30 (dd, J = 6.9, 3.9 Hz, 6H), 1.08 – 0.93 (m, 7H), 0.92 – 0.71 (m, 14H).^{13}C NMR (101 MHz, DMSO) δ 172.8, 170.2, 169.2, 168.0, 156.2, 155.8, 145.3, 144.1, 142.8, 133.1, 128.22, 128.18, 127.14, 127.10, 126.89, 126.85, 120.0, 119.9, 85.9, 82.1, 78.1, 77.4, 75.2, 67.4, 63.9, 63.8, 61.4, 60.7, 59.1, 58.6, 57.6, 56.3, 55.5, 54.65, 54.56, 50.2, 49.6, 47.7, 46.7, 45.8, 45.4, 44.2, 43.7, 37.6, 35.6, 32.2, 32.0, 30.3, 30.2, 27.3, 27.2, 25.8, 24.8, 23.6, 19.5, 19.3, 19.2, 19.0, 18.7, 17.1, 16.3, 16.1, 15.9, 15.7, 15.4, 10.9, 10.7. HRMS (ESI): m/z [M + H] calcd. for $C_{48}H_{76}N_7O_{11}S_2$: 990.5044 Found: 990.5034.

5. ^{1}H NMR Spectra

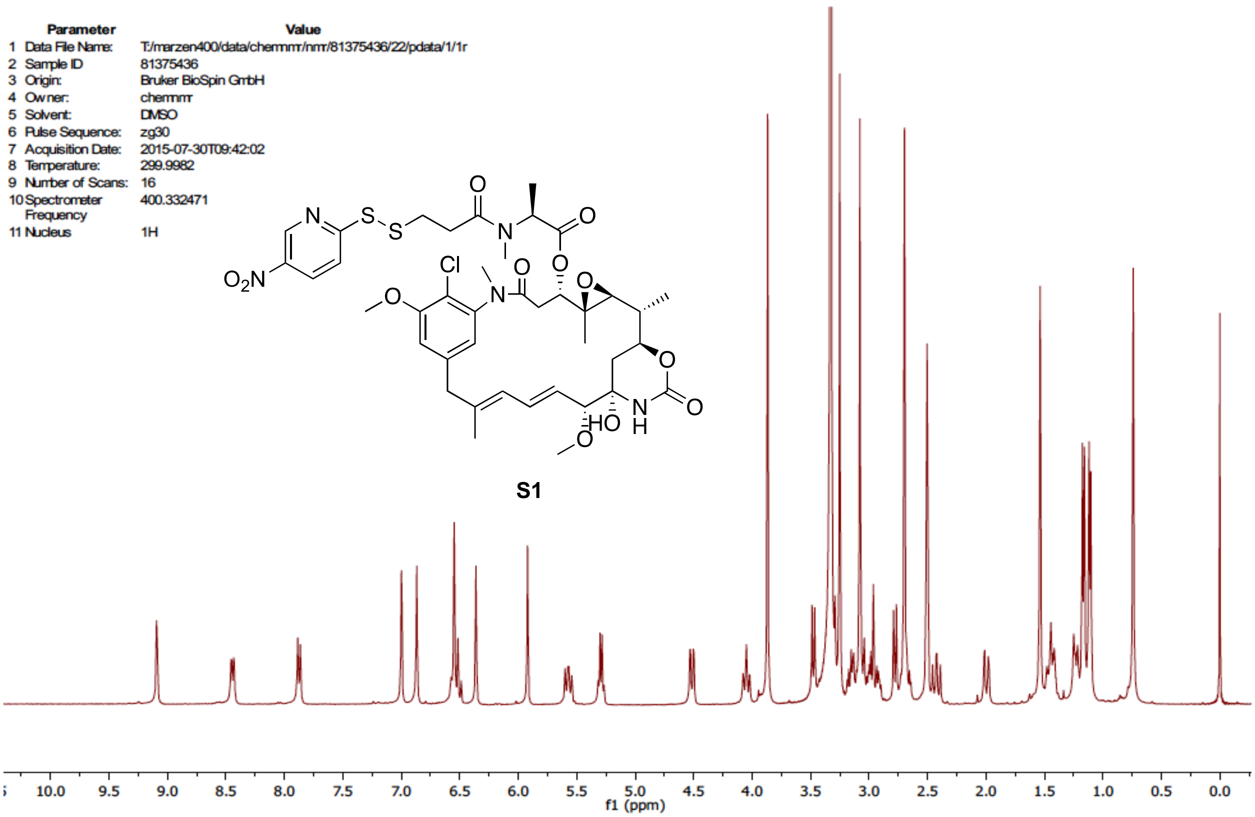
Parameter Value
1 Data File Name: T:/marzen400/data/chemmr/nmr/81375436/22/pdata/1/1r
2 Sample ID 81375436
3 Origin: Bruker BioSpin GmbH
4 Owner: chemmr
5 Solvent: DMSO
6 Pulse Sequence: zg30
7 Acquisition Date: 2015-07-30T09:42:02
8 Temperature: 299.9982
9 Number of Scans: 16
10 Spectrometer Frequency 400.332471
11 Nucleus 1H
O2N
N
S
Cl
O
HO
N
H
10.0 9.5 9.0 8.5 8.0 7.5 7.0 6.5 6.0 5.5 5.0 4.5 4.0 3.5 3.0 2.5 2.0 1.5 1.0 0.5 0.0
f1 (ppm)

S1

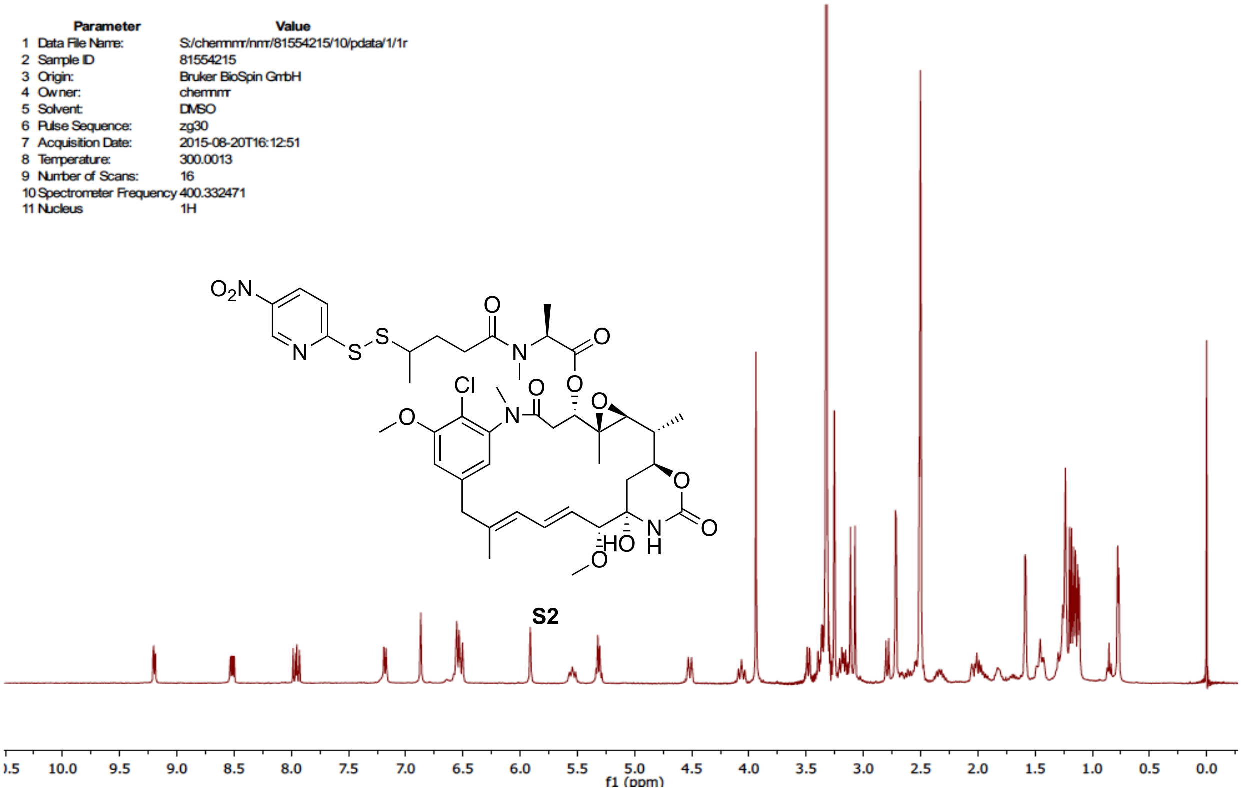
Parameter Value
1 Data File Name: S:/chemmr/nmr/81554215/10/pdata/1/1r
2 Sample ID 81554215
3 Origin: Bruker BioSpin GmbH
4 Owner: chemmr
5 Solvent: DMSO
6 Pulse Sequence: zg30
7 Acquisition Date: 2015-08-20T16:12:51
8 Temperature: 300.0013
9 Number of Scans: 16
10 Spectrometer Frequency 400.332471
11 Nucleus 1H
O2N
N
S
Cl
O
HO
N
H
10.0 9.5 9.0 8.5 8.0 7.5 7.0 6.5 6.0 5.5 5.0 4.5 4.0 3.5 3.0 2.5 2.0 1.5 1.0 0.5 0.0
f1 (ppm)

S2

	Parameter	Value
1	Data File Name:	T:/marzen400/data/chemmr/nmr/81372021/22/pdata/1/1r
2	Sample ID	81372021
3	Origin:	Bruker BioSpin GmbH
4	Owner:	chemmr
5	Solvent:	DMSO
6	Pulse Sequence:	zg30
7	Acquisition Date:	2015-07-30T10:00:00
8	Temperature:	299.9978
9	Number of Scans:	16
10	Spectrometer Frequency	400.332471
11	Nucleus	1H

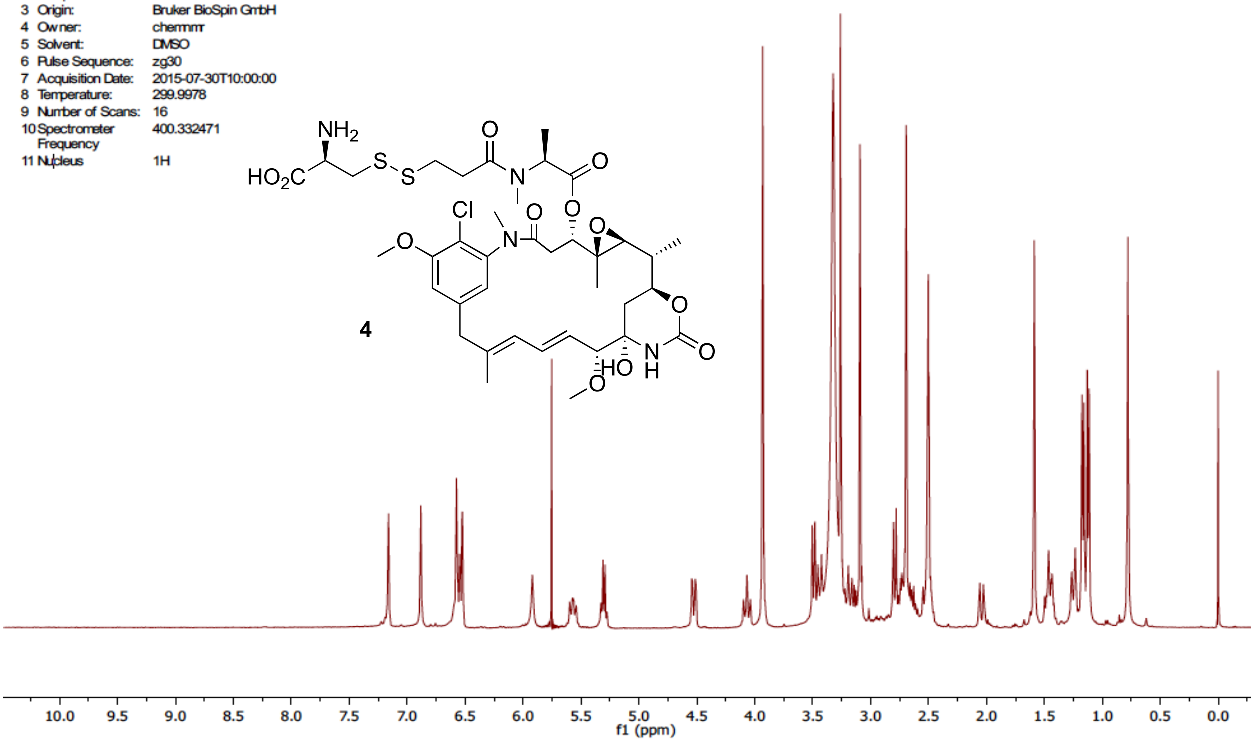

4

	Parameter	Value
1	Data File Name:	T:/NMR2012/data/liuy55/nmr/81554216/10/pdata/1/1r
2	Sample ID	81554216
3	Origin:	Bruker BioSpin GmbH
4	Owner:	smapchemist
5	Solvent:	DMSO
6	Pulse Sequence:	zg30
7	Acquisition Date:	2015-08-25T00:02:58
8	Temperature:	300
9	Number of Scans:	16
10	Spectrometer Frequency	500.2630016
11	Nucleus	1H

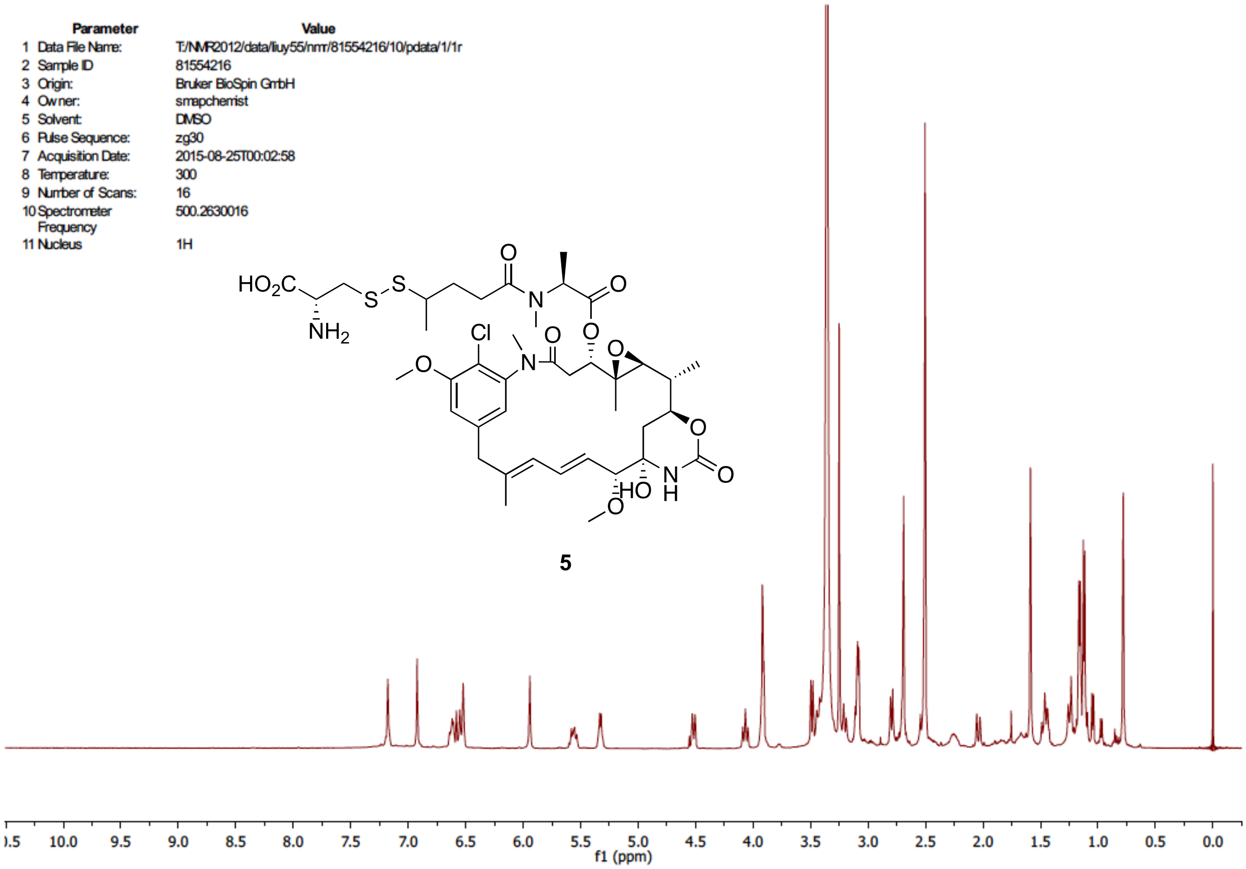

5

Parameter		Value
1	Data File Name:	Y:/thomashp/nmr/68778-SPDB/10/pdata/1/1r
2	Sample ID	68778-SPDB
3	Origin:	Bruker BioSpin GmbH
4	Owner:	chemnmr
5	Solvent:	DMSO
6	Pulse Sequence:	zg30
7	Acquisition Date:	2015-09-23T15:02:58
8	Temperature:	300
9	Number of Scans:	16
10	Spectrometer Frequency	400.132470966543
11	Nucleus	1H

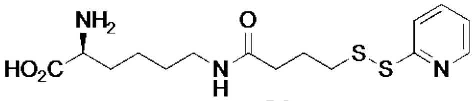

S4

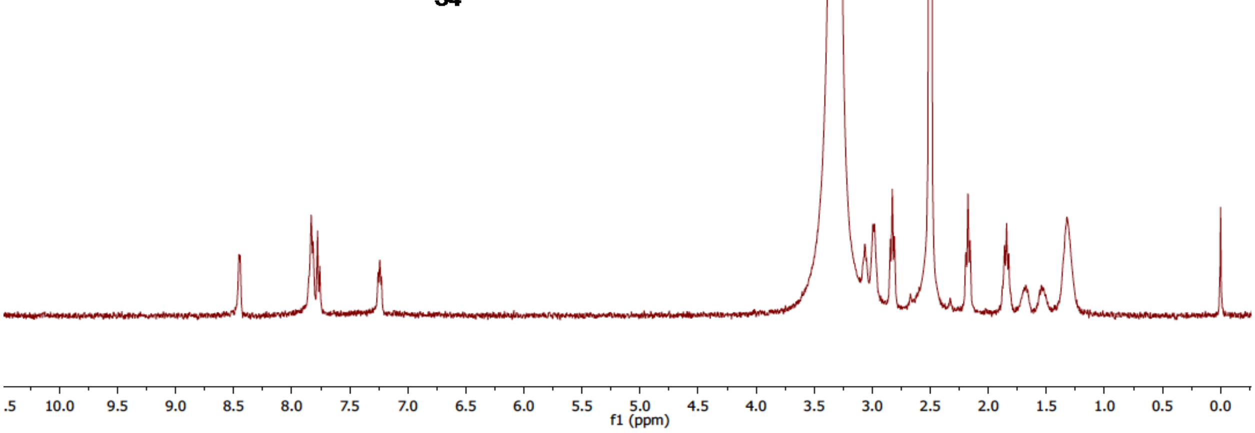

Parameter		Value
1	Data File Name:	T:/marzen400/data/chemnmr/nmr/81394538/22/pdata/1/1r
2	Sample ID	81394538
3	Origin:	Bruker BioSpin GmbH
4	Owner:	chemnmr
5	Solvent:	DMSO
6	Pulse Sequence:	zg30
7	Acquisition Date:	2015-07-30T09:56:24
8	Temperature:	299.9998
9	Number of Scans:	16
10	Spectrometer Frequency	400.332471
11	Nucleus	1H

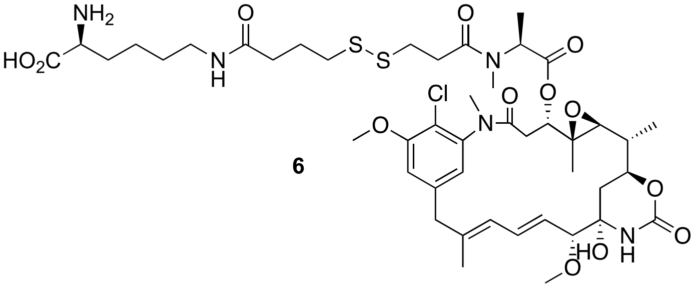

6

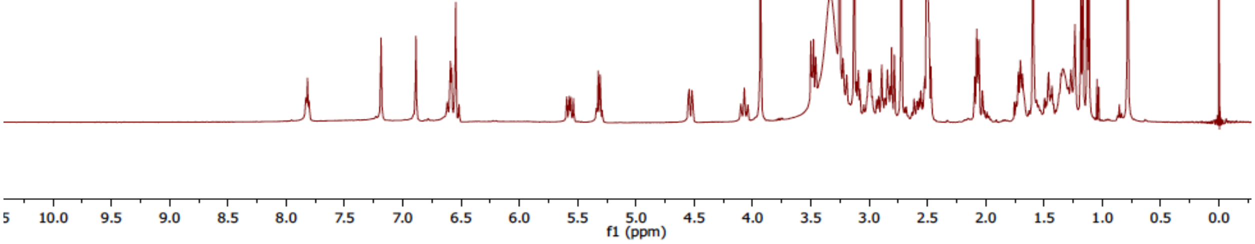

	Parameter	Value
1	Data File Name:	T:/marzen400/data/chemmr/nmr/81386765/22/pdata/1/1r
2	Sample ID	81386765
3	Origin:	Bruker BioSpin GmbH
4	Owner:	chemmr
5	Solvent:	DMSO
6	Pulse Sequence:	zg30
7	Acquisition Date:	2015-07-30T09:49:12
8	Temperature:	300.0028
9	Number of Scans:	16
10	Spectrometer Frequency	400.332471
11	Nucleus	1H

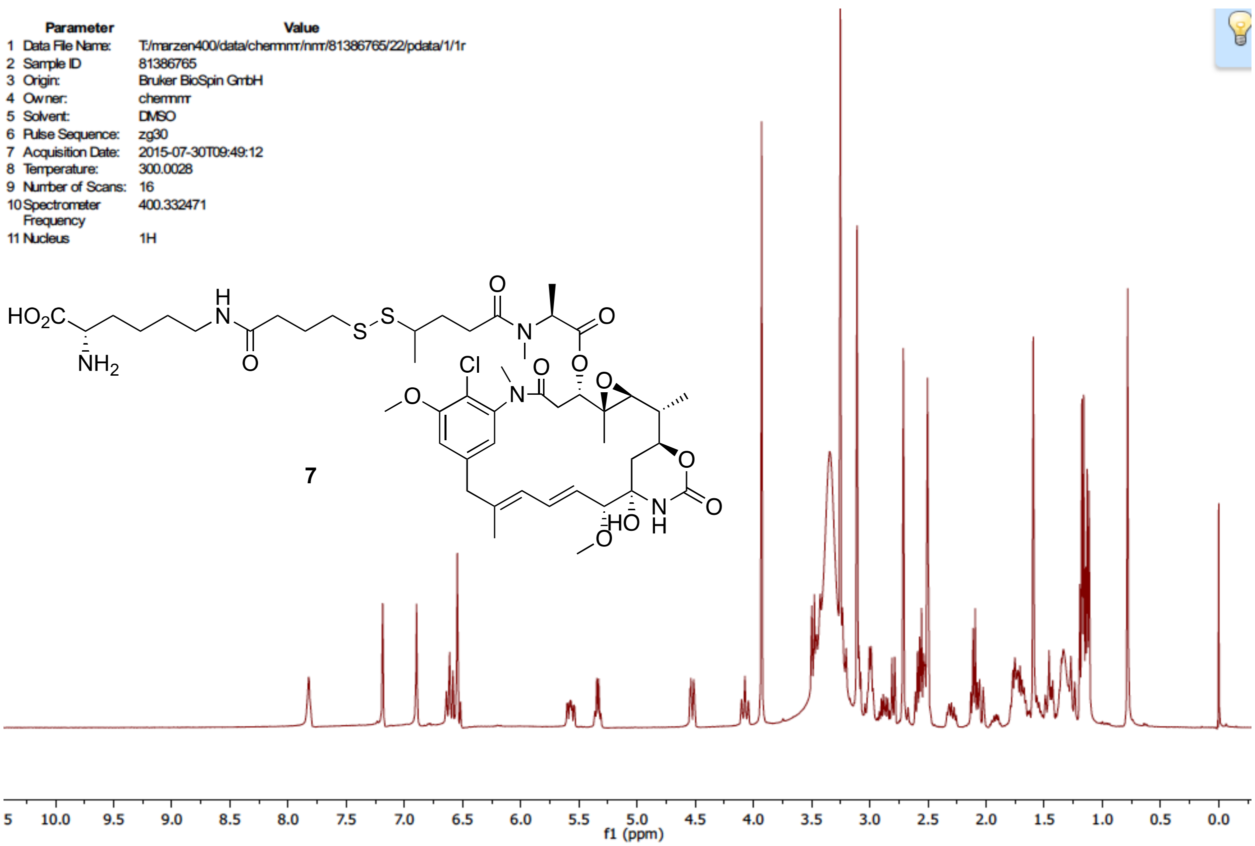

	Parameter	Value
1	Data File Name:	T:/marzen400/data/chemmr/nmr/81394537/22/pdata/1/1r
2	Sample ID	81394537
3	Origin:	Bruker BioSpin GmbH
4	Owner:	chemmr
5	Solvent:	DMSO
6	Pulse Sequence:	zg30
7	Acquisition Date:	2015-07-30T09:52:49
8	Temperature:	300.0001
9	Number of Scans:	16
10	Spectrometer Frequency	400.332471
11	Nucleus	1H

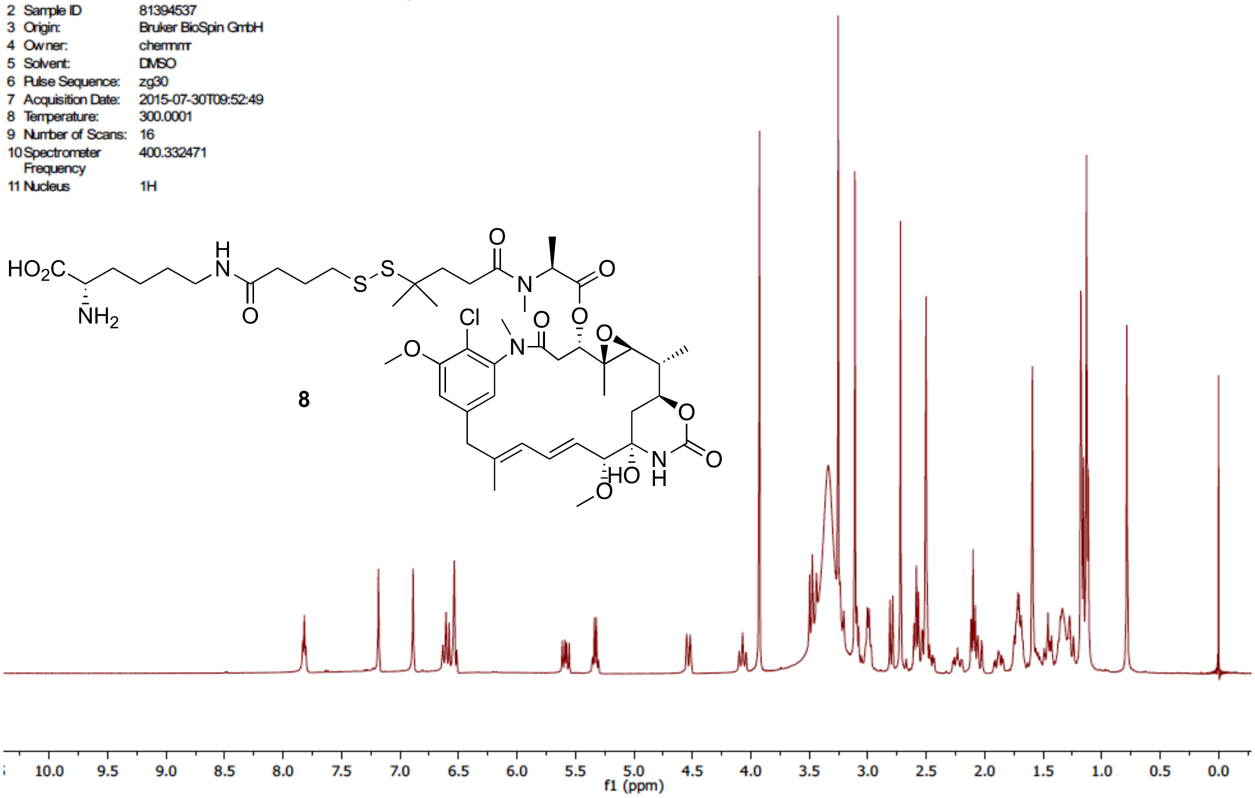

	Parameter	Value
1	Data File Name:	Y:/thomashp/nmr/68778-PDS-alcohol/10/pdata/1/1r
2	Sample ID	68778-PDS-alcohol
3	Origin:	Bruker BioSpin GmbH
4	Owner:	chemnmr
5	Solvent:	DMSO
6	Pulse Sequence:	zg30
7	Acquisition Date:	2015-09-23T14:19:36
8	Temperature:	300
9	Number of Scans:	16
10	Spectrometer Frequency	400.132470966543
11	Nucleus	1H

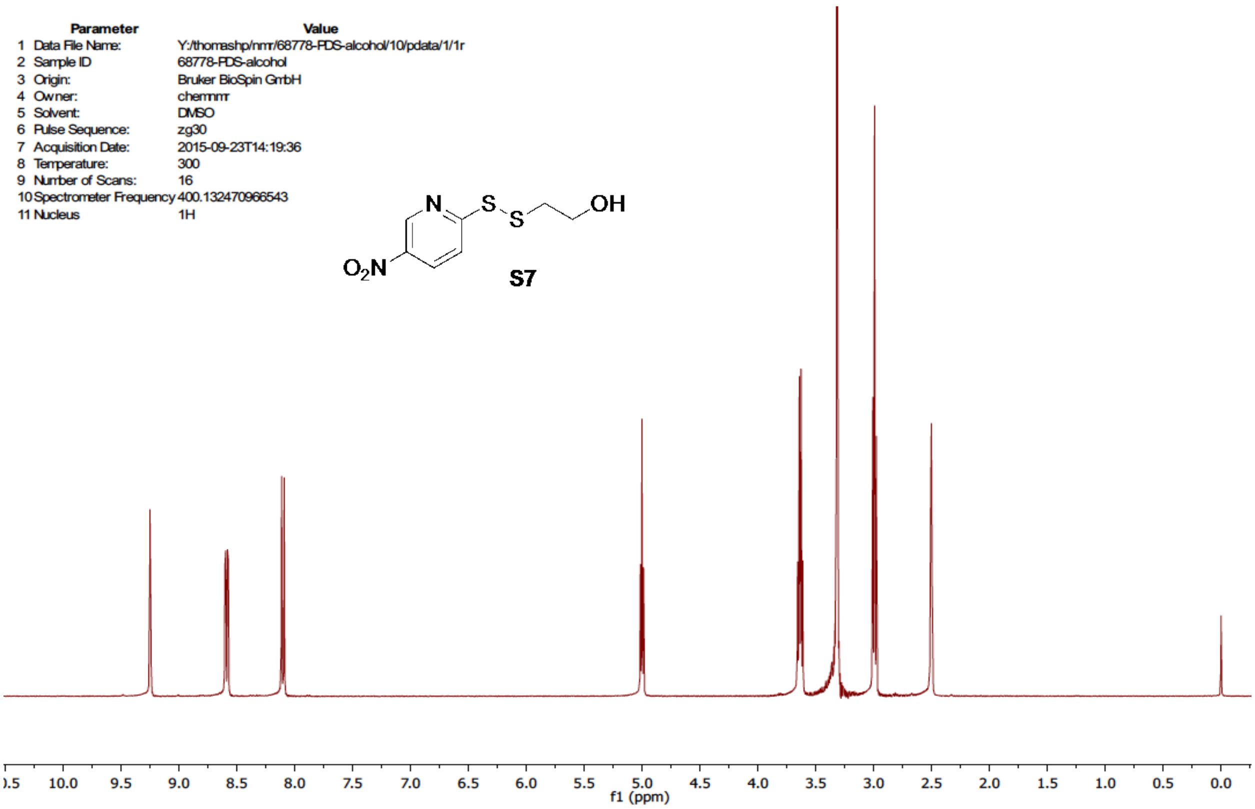

	Parameter	Value
1	Data File Name:	Y:/thomashp/nmr/G03068703/10/pdata/1/1r
2	Sample ID	G03068703
3	Origin:	Bruker BioSpin GmbH
4	Owner:	chemnmr
5	Solvent:	DMSO
6	Pulse Sequence:	zg30
7	Acquisition Date:	2015-08-31T18:30:22
8	Temperature:	300
9	Number of Scans:	32
10	Spectrometer Frequency	400.132470966543
11	Nucleus	1H

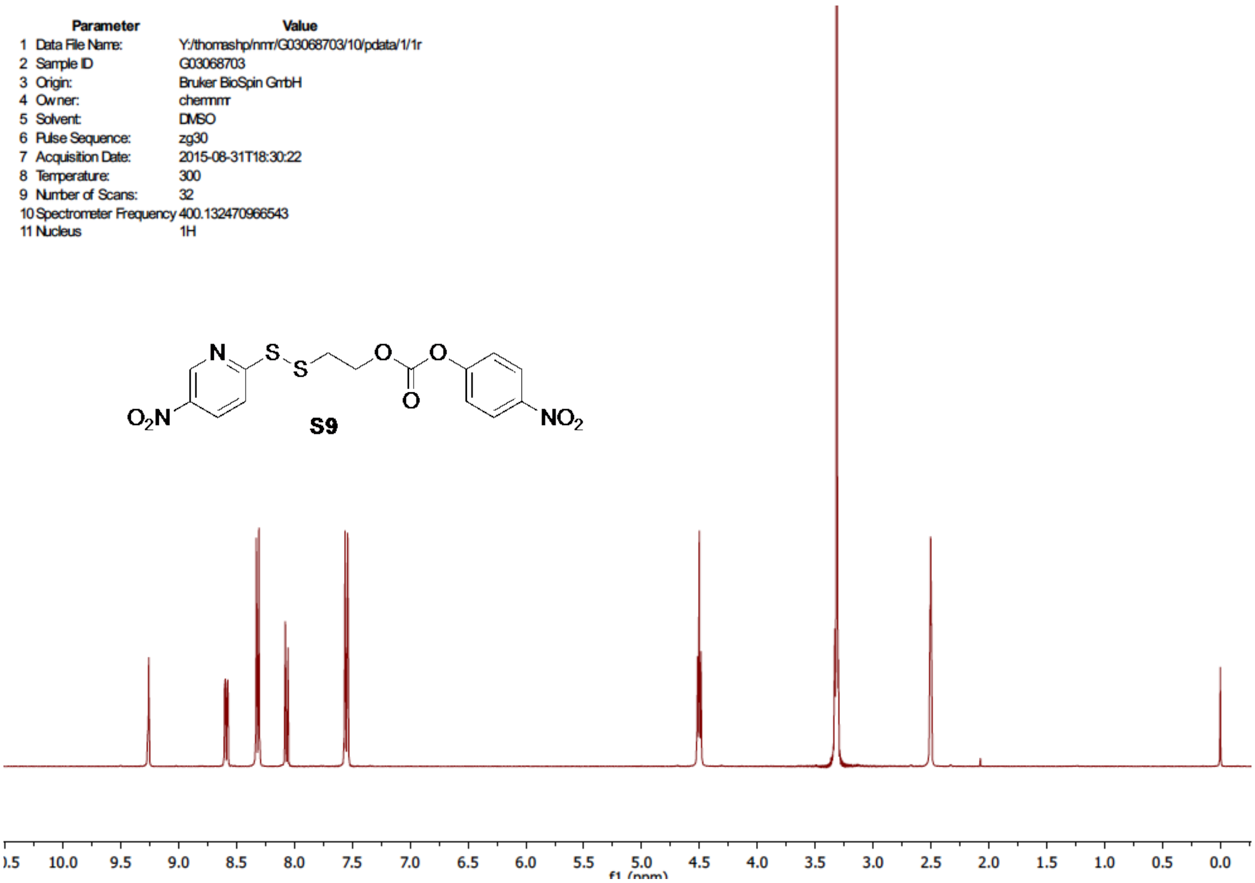

	Parameter	Value
1	Data File Name:	T:/marzen400/data/chemmr/nmr/80979040/13/pdata/1/1r
2	Sample ID	80979040
3	Origin:	Bruker BioSpin GmbH
4	Owner:	chemmr
5	Solvent:	Dioxane
6	Pulse Sequence:	zg30
7	Acquisition Date:	2015-08-21T11:30:17
8	Temperature:	299.9997
9	Number of Scans:	16
10	Spectrometer Frequency	400.332471
11	Nucleus	1H

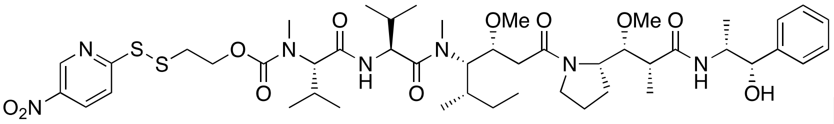

S11

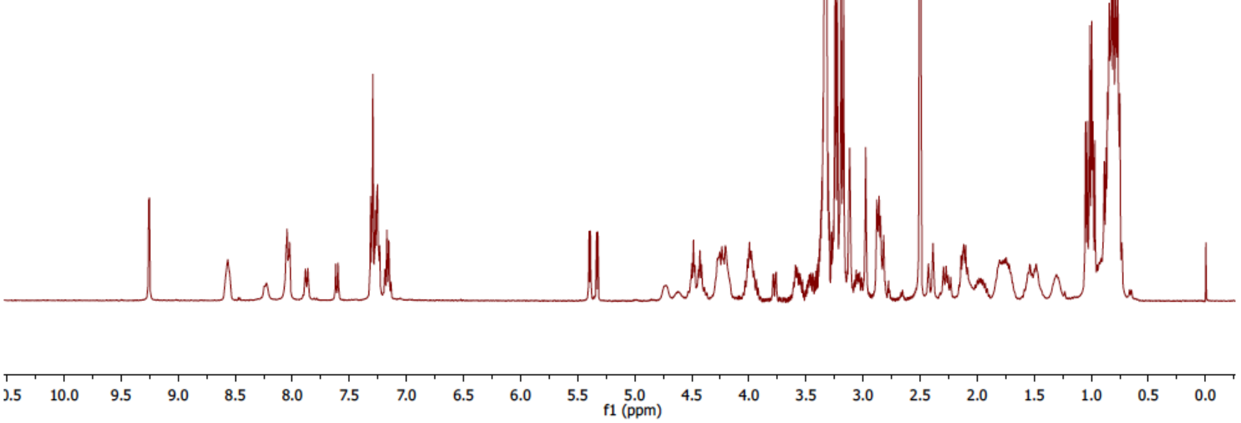

	Parameter	Value
1	Data File Name:	Y:/thomashp/nmr/G03068548/10/pdata/1/1r
2	Sample ID	G03068548
3	Origin:	Bruker BioSpin GmbH
4	Owner:	chemmr
5	Solvent:	DMSO
6	Pulse Sequence:	zg30
7	Acquisition Date:	2015-08-31T18:54:43
8	Temperature:	300
9	Number of Scans:	32
10	Spectrometer Frequency	400.132470966543
11	Nucleus	1H

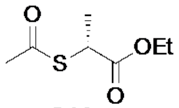

S14

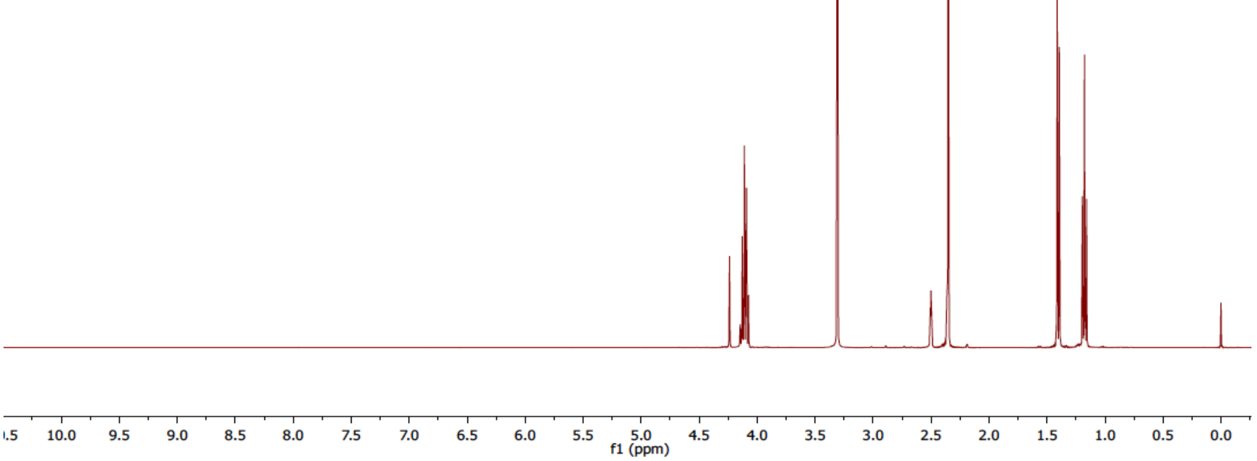

Parameter	Value
1 Data File Name:	Y:/thomashp/nmr/G03068915/10/pdata/1/1r
2 Sample ID	G03068915
3 Origin:	Bruker BioSpin GmbH
4 Owner:	chemnmr
5 Solvent:	DMSO
6 Pulse Sequence:	zg30
7 Acquisition Date:	2015-08-31T18:44:54
8 Temperature:	300
9 Number of Scans:	128
10 Spectrometer Frequency	400.132470966543
11 Nucleus	1H

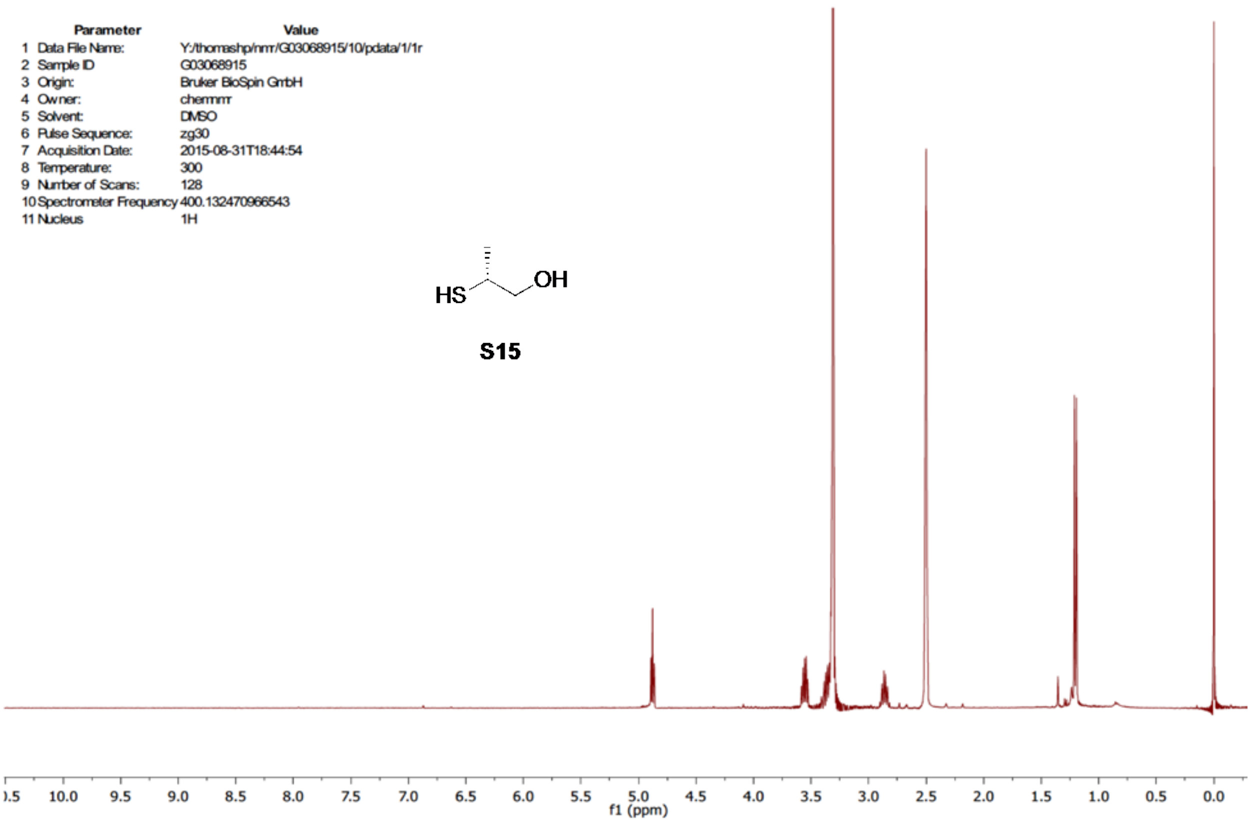

S15

Parameter	Value
1 Data File Name:	Y:/thomashp/nmr/G02941229/10/pdata/1/1r
2 Sample ID	G02941229
3 Origin:	Bruker BioSpin GmbH
4 Owner:	chemnmr
5 Solvent:	DMSO
6 Pulse Sequence:	zg30
7 Acquisition Date:	2015-08-31T18:35:12
8 Temperature:	300
9 Number of Scans:	32
10 Spectrometer Frequency	400.132470966543
11 Nucleus	1H

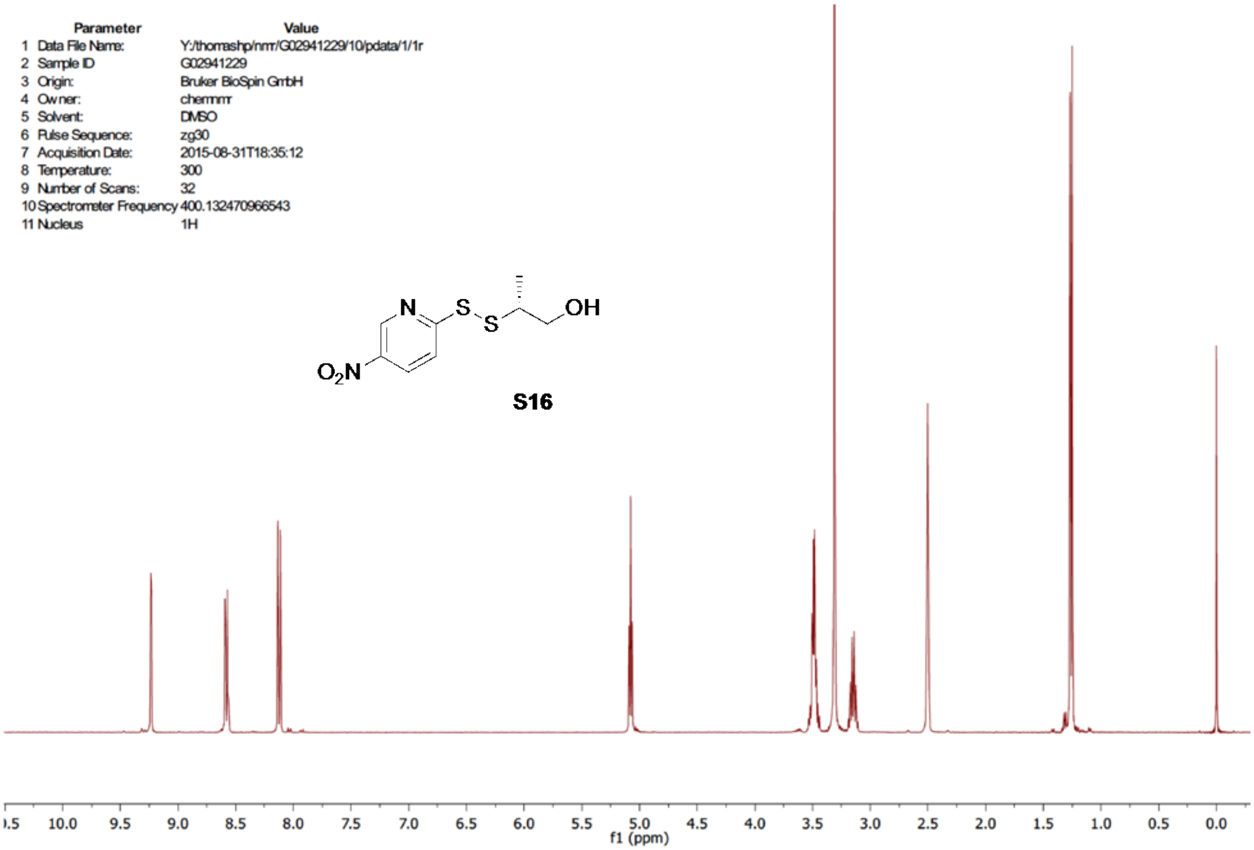

S16

	Parameter	Value
1	Data File Name:	Y:/thomashp/nmr/G03068703/10/pdata/1/1r
2	Sample ID	G03068703
3	Origin:	Bruker BioSpin GmbH
4	Owner:	chemnmr
5	Solvent:	DMSO
6	Pulse Sequence:	zg30
7	Acquisition Date:	2015-08-31T18:30:22
8	Temperature:	300
9	Number of Scans:	32
10	Spectrometer Frequency	400.132470966543
11	Nucleus	1H

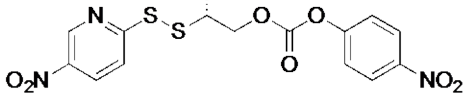

S17

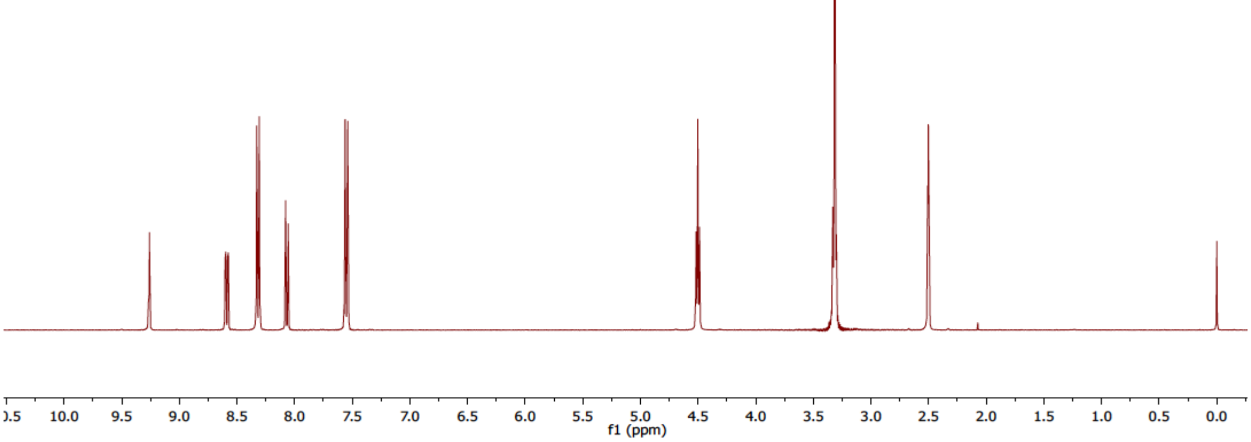

	Parameter	Value
1	Data File Name:	T:/marzen400/data/chemnmr/nmr/81166642/20/pdata/1/1r
2	Sample ID	81166642
3	Origin:	Bruker BioSpin GmbH
4	Owner:	chemnmr
5	Solvent:	DMSO
6	Pulse Sequence:	zg30
7	Acquisition Date:	2015-07-28T15:38:42
8	Temperature:	300.0014
9	Number of Scans:	32
10	Spectrometer Frequency	400.332471
11	Nucleus	1H

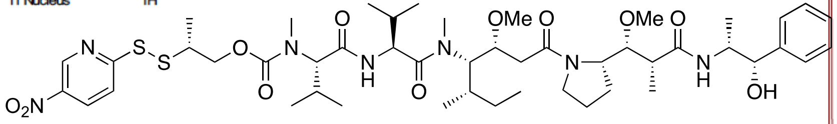

S18

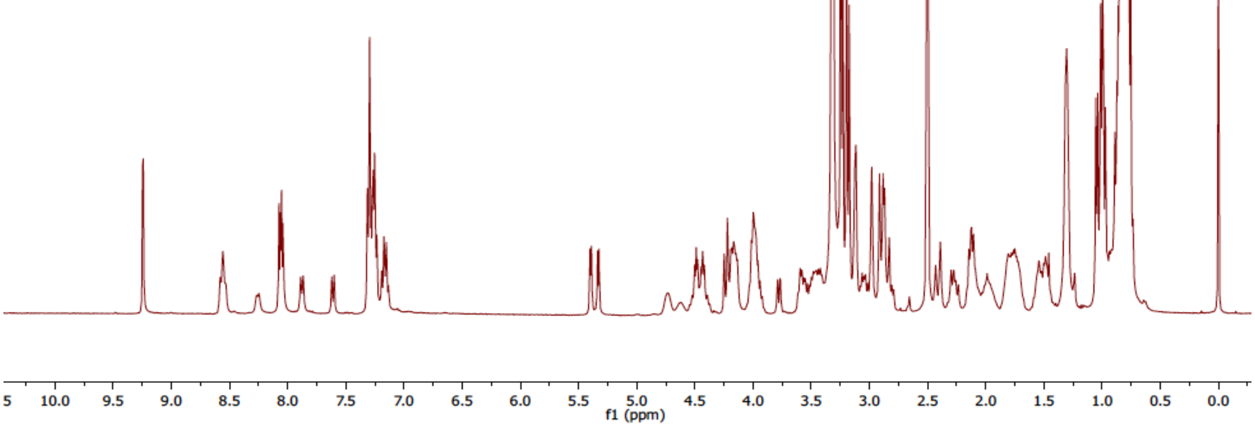

Supplement: Supplementary file 1 [file SC-008-C6SC01831A-s001.pdf]
